# Deciphering sevoflurane-induced neurotoxicity: from isolated targets to intricate regulatory networks

**DOI:** 10.3389/fnins.2025.1646246

**Published:** 2025-08-15

**Authors:** Yue Shu, Liang Bai, Shouyang Yu, Yulan Li

**Affiliations:** ^1^The First School of Clinical Medicine, Lanzhou University, Lanzhou, China; ^2^Physiology Teaching and Research Office, Zunyi Medical and Pharmaceutical College, Zunyi, China; ^3^Key Laboratory of Brain Science, Key Laboratory of Anesthesia and Organ Protection of Ministry of Education (In Cultivation), Zunyi Medical University, Zunyi, China; ^4^Department of Anesthesiology, The First Hospital of Lanzhou University, Lanzhou, China

**Keywords:** sevoflurane, ferroptosis, endoplasmic reticulum stress, neuroinflammation, BDNF, apoptosis

## Abstract

Among the 321 million surgeries performed globally each year, sevoflurane dominates the inhaled anesthesia field due to its unique pharmacological properties. However, studies indicate that sevoflurane exerts multiple adverse effects on the nervous system, and its potential neurotoxic effects are increasingly drawing attention. This article integrates multi-level evidence from molecular mechanisms, cellular models, animal experiments, and clinical studies to comprehensively elucidate the key mechanisms underlying sevoflurane-induced neurotoxicity, including ferroptosis pathway activation, calcium homeostasis disruption, BDNF signaling abnormalities, neuroinflammatory responses, and endoplasmic reticulum stress. The findings aim to provide a theoretical foundation for developing precise neuroprotective strategies and optimizing clinical anesthesia protocols.

## Graphical Abstract

Mechanisms of sevoflurane neurotoxicity. ATF4, Activating Transcription Factor 4; BDNF, Brain-Derived Neurotrophic Factor; CAP, Cholinergic Anti-inflammatory Pathway; eIF2α, Eukaryotic Initiation Factor 2α; IP3R, Inositol 1,4,5-TrisPhosphate Receptor; L-VGCCs, L-type Voltage-Gated Calcium Channels; mBDNF, Mature BDNF; NLRP1, NACHT, LRR, and PYD Domains-Containing Protein 1; PERK, PKR-like ER Kinase; p-IP3R, Phosphorylated IP3R; ProBDNF, Proprotein form of BDNF; PTP1B, Protein Tyrosine Phosphatase 1B; Rab1A, Ras-associated binding protein 1A; RyR, Ryanodine Receptor; Sev, Sevoflurane; SERCA2a, Sarco/Endoplasmic Reticulum Ca^2+^-ATPase 2a; TLR4, Toll-Like Receptor 4; TrkB, Tropomyosin receptor kinase B; T-VGCCs, T-type Voltage-Gated Calcium Channels. 
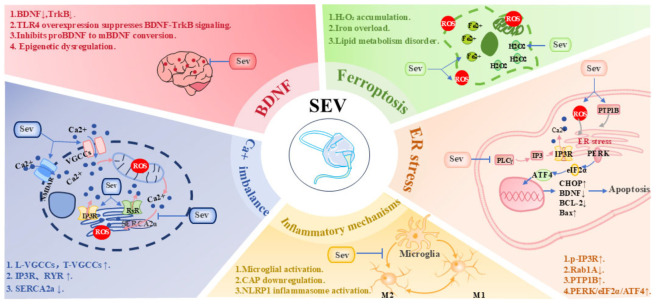

## 1 Introduction

Approximately 321 million surgical procedures are performed globally each year, with general anesthesia predominantly achieved through volatile anesthetic agents (Weiser et al., 2015). Sevoflurane has emerged as the dominant choice in inhaled anesthesia due to its distinctive pharmacological properties (Wu et al., 2025). However, as its clinical application continues to expand, growing concerns have been raised regarding its potential safety issues, particularly its age-related neurotoxicity and other adverse effects, which have become a major research focus.

### 1.1 Preclinical evidence for sevoflurane neurotoxicity

Based on current evidence from animal studies, sevoflurane anesthesia has been demonstrated to exert detrimental effects on both early neurodevelopment in rodents and cognitive function in aged models. Experimental research has confirmed sevoflurane induces cognitive impairment in neonatal mice by suppressing hippocampal neurogenesis (Zuo et al., 2022), and accelerates disease progression in Alzheimer’s disease model mice through mitochondrial fission (He et al., 2024). The adverse neurological effects of sevoflurane are mediated through multiple pathological pathways. In the developing brain, sevoflurane may disrupt normal neuronal differentiation, migration, and synaptogenesis, while activating apoptotic signaling pathways that induce neuronal apoptosis, ultimately impairing the normal development of cognitive functions such as learning and memory (Fang et al., 2017; Liu et al., 2016; Liu et al., 2017; Zeng et al., 2022). In the mature nervous system, sevoflurane disrupts the balance of neurotransmitter systems by interfering with the normal function of γ-aminobutyric acid (GABA), glutamate, and other neurotransmitters, leading to abnormal neuronal electrical activity and subsequent cognitive dysfunction (Wang et al., 2025). Although animal studies have provided crucial insights into the mechanisms of sevoflurane neurotoxicity, the clinical relevance of these findings requires further validation through rigorously designed human studies.

### 1.2 Sevoflurane neurotoxicity in humans

Recent clinical trials have sought to address the translational gap in sevoflurane neurotoxicity.

The PANDA and GAS studies demonstrated that a single, brief sevoflurane anesthesia exposure (<1 h) did not significantly affect neurodevelopment in infants and young children (<5 years old) (McCann et al., 2019; McCann et al., 2019; Sun et al., 2016), whereas the MASK trial focused on cumulative effects, suggesting that multiple exposures (≥3) were associated with a modest increase in the risk of learning disabilities (Warner et al., 2018). Another retrospective study similarly indicated that multiple anesthesia exposures (including sevoflurane) before age 3 are associated with a modest elevation in the risk of subsequent learning disabilities and behavioral disorders, such as ADHD (DiMaggio et al., 2011).

In elderly patients, cognitive impairment was observed in 57% and 9% of cases at 1 and 3 h post-sevoflurane anesthesia, respectively (Chen et al., 2001). This phenomenon is considered to be associated with post-anesthesia cortical thinning in brain regions implicated in Alzheimer’s disease (Sprung et al., 2019). Moreover, advanced age (≥60 years) serves as an independent risk factor for postoperative cognitive dysfunction (POCD) at 3 months, conferring significant risks of long-term cognitive impairment in elderly patients (Monk et al., 2008). An ongoing randomized controlled study (NCT03326960) is being conducted to investigate the effects of sevoflurane, desflurane, and propofol anesthesia on neurocognitive outcomes (such as postoperative delirium and postoperative cognitive dysfunction) and corresponding biomarker profiles (RNFL-C, RNFL-T) in elderly patients (ClinicalTrials.gov., 2020). Another ongoing observational study (NCT05991817) is investigating the dynamic changes in spatial memory and plasma inflammatory cytokine profiles in patients undergoing elective surgery before and after sevoflurane anesthesia. This study aims to elucidate the potential mechanisms underlying sevoflurane-associated delayed neurocognitive recovery, particularly spatial memory impairment, in middle-aged individuals (ClinicalTrials.gov., 2024).

Notwithstanding the current limitations in clinical evidence concerning both sevoflurane-induced neurotoxic effects and mitigation strategies in human subjects - primarily reflected in inadequate sample sizes and insufficient long-term follow-up data - the systematic elucidation of its neurotoxic characteristics and underlying molecular mechanisms carries imperative clinical significance, given its extensive worldwide utilization in clinical practice. This narrative review summarizes key findings without systematic search or meta-analysis, selectively integrating representative multidisciplinary evidence to provide a conceptual framework for understanding sevoflurane-induced neurotoxicity. Through illustrative examples spanning molecular pathways, cellular models, animal studies, and clinical observations, we contextualize current insights to advance mechanistic hypotheses, highlight potential neuroprotective strategies, and stimulate further investigation into optimizing anesthesia protocols for vulnerable populations.

## 2 Mechanisms of neurotoxicity

### 2.1 Ferroptosis

**FIGURE 1:**
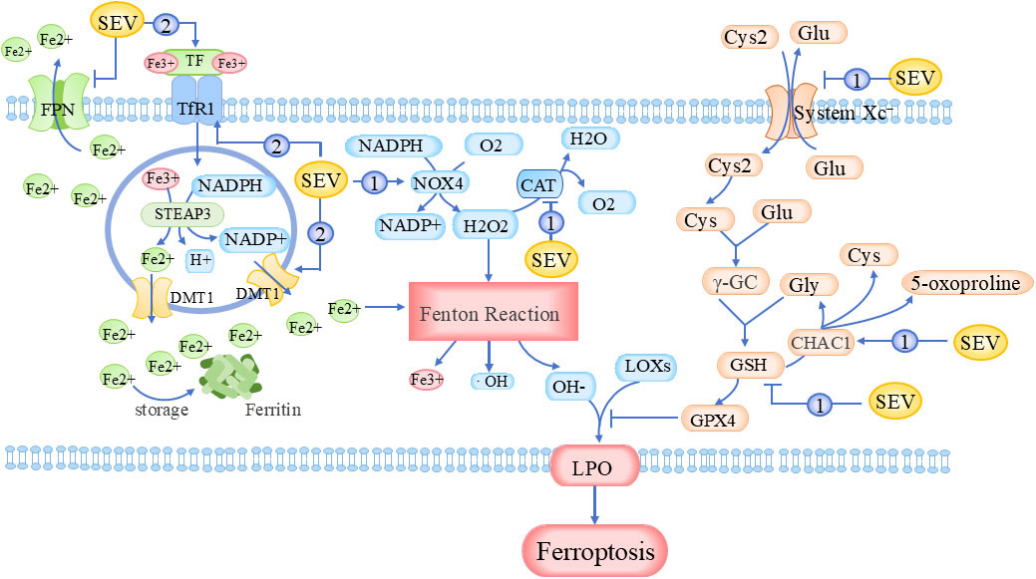
Mechanisms of sevoflurane-induced ferroptosis. CAT, Catalase; CHAC1, ChaC Glutathione-Specific γ-Glutamylcyclotransferase 1; Cys2, Cystine; DMT1, Divalent Metal Transporter 1; FPN, Ferroportin; Glu, Glutamate; Gly, Glycine; LOXs, Lipoxygenases; LPO, Lipid Peroxidation; NADPH, Nicotinamide Adenine Dinucleotide Phosphate; NOX4, NADPH Oxidase 4; STEAP3, Six-Transmembrane Epithelial Antigen of Prostate 3; System Xc^–^, Cystine/Glutamate Antiporter; TfR1, Transferrin Receptor 1; γ-GC, γ-Glutamylcysteine.

Ferroptosis was first proposed in 2012 by the Brent R. Stockwell laboratory (Dixon et al., 2012). It is a form of regulated cell death characterized by iron-dependent lipid peroxidation, driven by hydrogen peroxide (H_2_O_2_) and ferrous iron (Fe^2+^) through the Fenton reaction. The resulting reactive oxygen species (ROS) oxidize polyunsaturated fatty acids (PUFAs), which disrupt cell membrane integrity and ultimately induce cell death. This process is tightly regulated by iron metabolism, antioxidant defense systems, and lipid metabolic pathways, and is closely associated with neurodegenerative diseases, ischemia-reperfusion injury, and cancer therapeutics. In neonatal mice at approximately 1 week of age, studies have shown that sevoflurane enhances cellular ferroptosis in a concentration-dependent manner (Table 1) (Zhang P. et al., 2022). Specifically, sevoflurane compromises the oxidative stress defense system by activating transcription factors ATF3 and ATF4 through endoplasmic reticulum stress (ERS) pathways. This subsequently suppresses the nuclear factor erythroid 2-related factor 2 (Nrf2)-mediated antioxidant defense system while upregulating pro-oxidant molecules NOX4 and ChaC glutathione-specific γ-glutamylcyclotransferase 1 (CHAC1). Concurrently, sevoflurane inhibits the expression of antioxidant proteins including glutathione (GSH), catalase, and SLC7A11 (Kang et al., 2024; Li P. et al., 2025; Xu et al., 2022; Xu et al., 2023), ultimately leading to abnormal intracellular accumulation of lipid peroxides and H_2_O_2_ (Figure 1, Loop 1). Regarding iron metabolism, sevoflurane upregulates the expression of transferrin receptor (TfR), transferrin (TF), and divalent metal transporter 1 (DMT1), thereby enhancing cellular iron uptake and accelerating its cytosolic translocation. Concurrently, it downregulates ferroportin (FPN) expression, reducing intracellular iron efflux, ultimately leading to cellular iron overload (Hu et al., 2024; Zhang et al., 2024; Figure 1, Loop 2). Liu’s study further demonstrated that sevoflurane mediates ferroptosis and myelin degeneration in neonatal mouse oligodendrocytes by inhibiting TfR1 palmitoylation and enhancing TfR1 endocytosis (Liu et al., 2025).

**TABLE 1:** Dose-age dependency in sevoflurane neurotoxicity.

Age group	Critical dose threshold	Minimum exposure duration	Primary mechanisms	Functional outcomes
Neonatal (postnatal days 6–8) mice	3% sevoflurane	2 h × 3 days	Mitochondrial dysfunction, Ferroptosis	Cognitive impairments (Zhang P. et al., 2022; Hu et al., 2024; Liu et al., 2025)
			Enhance TfR1 endocytosis	Hypomyelination (Liu et al., 2025)
			miR-182-5p↑, Iron overload, Lipid peroxide accumulation	Hearing impairment, Ribbon synapse damage (Jin et al., 2024)
			Enhance TfR1 endocytosis	Cognitive impairments, Hypomyelination (Liu et al., 2025)
			PRKCD↑, Hippo pathway↑, Ferroptosis	Hippocampal neuron injury Learning and memory dysfunction (Lv et al., 2024)
			L-VGCCs↑, Calcium overload	Cognitive impairment (Zeng et al., 2022)
			SIRT1↓,BNDF↓	Spatial memory deficits (Tang X. et al., 2020)
			SETD1B↓,NLRP1↑, caspase-1↑,GSDMD-N↑, IL-1β↑, IL-18↑	Cognitive impairment (Wang Z. et al., 2024)
Aged mice (18-months-old)	3% sevoflurane	2 h × 3 days	Ferroptosis, Mitochondrial Dysfunction	Cognitive dysfunction (Zeng et al., 2024)
			MIB2↑, Ferroptosis	Cognitive dysfunction (Zhao L. et al., 2021)
			NMDAR↑,RIPK1^p^↑ Calcium dyshomeostasis, necroptosis	Cognitive dysfunction (Liu et al., 2024)
		3 h × 3 days	NMDAR-NR2B↓	Memory Impairment (Tian et al., 2016)
Aged mice model	3.2% sevoflurane	6 h	TREM1↑,TNF-α↑,IL-1β↑, M1-polarized microglia↑	Perioperative, neurocognitive disorders (Tang et al., 2023)
Neonatal rats (postnatal day 6)	3% sevoflurane	2 h × 3 days	PAI-1↑,BDNF↓, TrkB ↓	Learning and memory dysfunction (Dong et al., 2020b)
			Histone deacetylation↑, BDNF↓	Neurodevelopmental deficits (Jia et al., 2016)
			PKA/CREB/BDNF↓	Adult-onset cognitive impairment (Zhao J. et al., 2021)
Neonatal rats (postnatal days 7–9)	2.3%	6 h	PTP1B↑,ERS↑	Neurodegeneration (Liu et al., 2019)
	3% sevoflurane	2 h × 3 days	DNA methyltransferases↑ BDNF hypermethylation↑ Reelin hypermethylation↑	Cognitive Impairments (Ju et al., 2016)
	3% sevoflurane	6 h	ATF4↑,NOX4↑, H_2_O_2_↑,	Spatial memory dysfunction (Liu et al., 2017)
Neonatal rats (postnatal days 9–11)	6% sevoflurane, 3% sevoflurane	6% × 6 min +3% × 9 min	T-VGCCs↑	Post-anesthetic hyperexcitatory behaviors (Shen et al., 2020)
Early-stage developing rats (week 1 to week 3)	3% sevoflurane	6 h	L-VGCCs↓	Neurodevelopmental impairments (Liu et al., 2016)
Pregnant rats (gestational day 18)	3.5% sevoflurane	/	TLR4↑, BDNF/TrkB/CREB↓	Spatial learning-memory impairment in rat offspring (Li Q. Q. et al., 2025)
Adult rats (2-months-old)	1.5% sevoflurane 3% sevoflurane	2 h	TNF-α,IL-1β,IL-6↑, Caspase-3↑	Dose-dependent cognitive dysfunction (Cui et al., 2018)
Aged rats (18-months-old)	2% sevoflurane	5 h	Tbx2↑, BDNF/Nrf2↓	Cognitive dysfunction, Hippocampal neuronal loss (Xu et al., 2023)
Aged rats (18-months-old)	2% sevoflurane	5 h	IP3Rs↑ ERS↑	Cognitive dysfunction (Zhang Q. et al., 2022)
	2.5% sevoflurane	6 h	Capn4↑, miR-124↓ NF-κB↓	Hippocampal neuronal apoptosis, Neuroinflammatory responses (Zhao Z. et al., 2021)
	3% sevoflurane	2 h	PERK/eIF2α/ATF4↑,GRP78↑,ERS↑	Cognitive dysfunction (Wang Y. et al., 2023)
	3.6% sevoflurane	6 h	NMDAR-NMNAT1/2↓	Neuronal inflammation cognitive impairmen (Yang et al., 2020)
	7% sevoflurane	3 h	RNA HOTAIR↑, Sin3A/coREST↑, REST↑,BNDF↓	Cognitive dysfunction (Wang et al., 2018)
Aged rats (32-months-old)	3% sevoflurane	3 h	acetylcholinesterase↑, TNF-α↑,	Cognitive impairments (Yin et al., 2019)

ATF4, Activating Transcription Factor 4; BNDF, Brain-derived neurotrophic factor; Capn4, Calpain 4 (regulatory subunit of calpains); CREB, cAMP Response Element-Binding protein; ERS, Endoplasmic Reticulum Stress; GRP78, Glucose-Regulated Protein 78 (HSPA5/BiP); H_2_O_2_, Hydrogen Peroxide; IL-1β, Interleukin-1 beta; IL-18, Interleukin-18; IP3Rs, Inositol 1,4,5-Trisphosphate Receptors; L-VGCCs, L-type voltage-gated calcium channels; MIB2, Mind bomb-2; NLRP1, NLR Family Pyrin Domain Containing 1; NMDAR, N-methyl-D-aspartate receptor; NOX4, NADPH Oxidase 4; PAI-1, Plasminogen Activator Inhibitor-1; PKA, Protein Kinase A; PRKCD, Protein kinase C delta; PTP1B, Protein Tyrosine Phosphatase 1B; REST, RE1-Silencing Transcription Factor; RIPK1, Receptor-Interacting Protein Kinase 1; SETD1B, SET domain containing 1B; SIRT1, Sirtuin 1; Tbx2, T-box Transcription Factor 2; TfR1, Transferrin receptor 1; TLR4, Toll-Like Receptor 4; TNF-α, Tumor necrosis factor-alpha; TREM1, Triggering receptor expressed on myeloid cells 1; TrkB, Tropomyosin Receptor Kinase B; T-VGCCs, T-type voltage-gated calcium channels.

Regarding lipid metabolism, perilipin 4 (PLIN4) protein participates in regulating lipid droplet formation and lipolysis processes. Sevoflurane induces lipid metabolic imbalance through PLIN4 overexpression, ultimately leading to iron-induced hippocampal neuronal death (Zeng et al., 2024). Glutathione Peroxidase 4 (GPX4), as a pivotal member of the glutathione peroxidase family, serves as the central regulator of ferroptosis. It specifically reduces lipid peroxides to maintain cellular membrane integrity, representing a core component of the cellular antioxidant defense system. Sevoflurane significantly inhibits GPX4 expression and function through multidimensional regulatory mechanisms, thereby synergistically promoting ferroptosis progression: (1) At the post-transcriptional level, sevoflurane specifically upregulates the expression of miR-182-5p in P7-P9 neonatal mice, which binds to the 3′-untranslated region (3′-UTR) of GPX4 mRNA through seed sequence complementarity, markedly inhibiting its translation efficiency (Jin et al., 2024); (2) At the protein stability level, sevoflurane activates the ubiquitin ligase MIB2 in aged mice (≥20-months-old), inducing polyubiquitination modification of GPX4 and thereby accelerating its degradation (Zhao L. et al., 2021); (3) In terms of transcriptional regulation, sevoflurane upregulates the expression of T-Box transcription factor 2 (Tbx2), inhibits the brain-derived neurotrophic factor (BDNF)-Nrf2 signaling axis, and downregulates GPX4 synthesis at the transcriptional level in aged rat model as illustrated in Table 1 (Xu et al., 2023). These three mechanisms form a cascade amplification effect, ultimately leading to the loss of intracellular GPX4 activity, accumulation of lipid peroxides, and triggering of iron-dependent cell death. In contrast to its pro-ferroptotic effects described above, sevoflurane has also been demonstrated to exhibit significant anti-ferroptotic activity through negative regulation of the key ferroptosis execution protein acyl-CoA synthetase long-chain family member 4 (ACSL4). Under physiological conditions, ACSL4 enhances membrane susceptibility to lipid peroxidation by catalyzing the esterification and membrane incorporation of PUFAs. However, sevoflurane downregulates ACSL4 expression at the transcriptional level by suppressing its critical transcription factor specificity protein 1 (SP1). This mechanism effectively maintains membrane stability and significantly mitigates ferroptosis induced by cerebral ischemia-reperfusion injury (Lyu and Li, 2023). These findings reveal sevoflurane’s unique pharmacological property of potentially exhibiting dual roles in ferroptosis regulation. Importantly, this bidirectional modulation (both pro-death and anti-death effects) may be influenced by pathological status, cell type, and exposure duration/dosage, with the net effect determined by the integrative coordination of these factors within the microenvironment.

Based on these mechanisms, several neuroprotective intervention strategies have been proposed: (1) Echinatin significantly improves intracellular iron homeostasis and enhances antioxidant capacity (You et al., 2024); (2) Melatonin increases neuronal survival rates by activating the Nrf2/GPX4 pathway (Ni H. et al., 2024); and (3) Targeted knockdown of Tbx2 or MIB2 reduces ferroptotic cell death (Xu et al., 2023; Zhao L. et al., 2021). Animal studies have further confirmed that these interventions effectively improve cognitive function and synaptic plasticity following sevoflurane exposure. These findings not only deepen our understanding of the neurotoxic mechanisms of anesthetic agents but also provide precise molecular targets and intervention strategies for the clinical prevention and treatment of perioperative neurocognitive disorders. Furthermore, the neuroprotective role of the Hippo signaling pathway has garnered increasing attention in the scientific community. The core effector molecule of this pathway, YAP1 (Yes-associated protein 1), has been shown to significantly inhibit ferroptosis progression, effectively alleviate cellular senescence, and improve cognitive dysfunction (Xu X. et al., 2021). Sevoflurane has been demonstrated to suppress the Hippo pathway through multiple targets, thereby inducing ferroptosis. Specifically, sevoflurane upregulates PLIN4 and PRKCD (protein kinase C delta) while inhibiting YAP1 expression. Targeted knockdown of PLIN4 and PRKCD has been shown to successfully mitigate sevoflurane-induced neurotoxicity and cognitive impairment (Lv et al., 2024; Zeng et al., 2024). Additionally, sevoflurane upregulates miR-144-3p, which directly targets and suppresses YAP1 expression, accelerating neuroblastoma apoptosis. However, this study did not explicitly investigate whether the miR-144-3p/YAP1 axis participates in regulating ferroptosis, leaving this mechanism to be verified in subsequent research.

Although current research on sevoflurane-induced neurotoxicity and ferroptosis remains limited, these pioneering findings provide novel theoretical perspectives for understanding the molecular mechanisms underlying inhalation anesthesia complications. They offer specific therapeutic targets for developing neuroprotective strategies based on ferroptosis inhibitors, potentially advancing new approaches for preventing and treating perioperative neurological complications.

### 2.2 Calcium dyshomeostasis

Calcium ions (Ca^2+^), as crucial secondary messengers in the central nervous system, not only regulate neurotransmitter release and synaptic plasticity but also participate in gene expression modulation and mitochondrial function maintenance. Calcium dyshomeostasis can lead to mitochondrial damage, impaired ATP synthesis, and ROS accumulation, thereby triggering apoptotic pathways (Zeng et al., 2022). Volatile anesthetics elevate intracellular calcium concentration ([Ca^2+^]i) in the hippocampus (Zeng et al., 2022), cerebral cortex (Chen K. et al., 2022), embryonic cortical neurons (Franks et al., 1998), and dorsal root ganglion neurons (Gomes et al., 2004) of rodents. This mechanism may be closely associated with their neurotoxic effects.

Cytosolic calcium concentration ([Ca^2+^]c) primarily originate from endoplasmic reticulum (ER) calcium store release and extracellular calcium influx. Cellular depolarization or ligand binding triggers Ca^2+^ influx through voltage-gated calcium channels (VGCCs) or N-methyl-D-aspartate receptor (NMDAR) channels, subsequently activating inositol 1,4,5-trisphosphate receptors (IP3Rs) and/or ryanodine receptors (RyRs) to induce calcium store release, leading to a significant increase in ([Ca^2+^]i). [Ca^2+^]i is actively transported back into the ER lumen via sarco/endoplasmic reticulum Ca^2+^-ATPase (SERCA) pumps in an ATP-dependent manner. Simultaneously, the sodium-calcium exchanger (NCX) on the plasma membrane utilizes the Na^+^ electrochemical gradient for bidirectional calcium transport, collectively maintaining the dynamic equilibrium of cellular calcium homeostasis (Ay et al., 2005).

**FIGURE 2:**
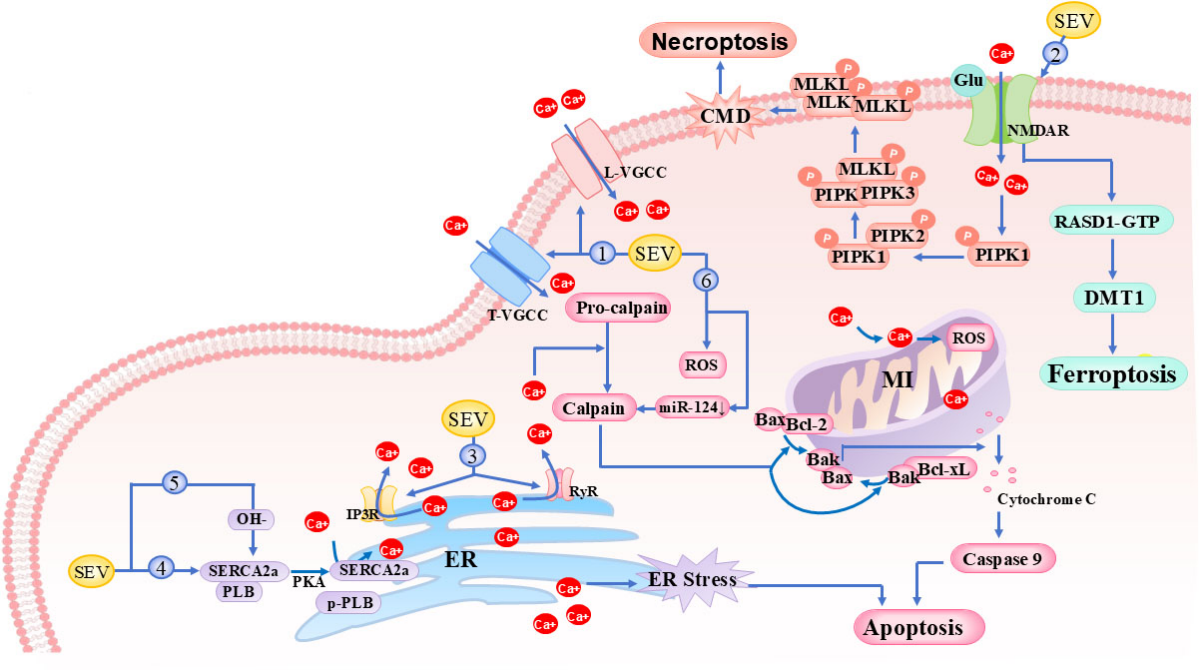
Mechanisms of sevoflurane-induced calcium dyshomeostasis. Bak, Bcl-2 antagonist/killer 1; Bax, Bcl-2-associated X protein; Bcl-2, B-cell lymphoma 2; CMD, Cell Membrane Damage; DMT1, Divalent Metal Transporter 1 (SLC11A2); ER, Endoplasmic Reticulum; Glu, Glutamate; IP3R, Inositol 1,4,5-TrisPhosphate Receptor; L-VGCC, L-type Voltage-Gated Calcium Channel; MLKL, Mixed Lineage Kinase Domain-Like; NMDAR, N-methyl-D-aspartate Receptor; PIPK, PhosphatidylInositol Phosphate Kinase; PKA, Protein Kinase A; PLB, Phospholamban; p-PLB, Phosphorylated Phospholamban; RASD1, RAS Dexamethasone-induced 1 (Dexras1); RyR, Ryanodine Receptor; SERCA2a, Sarco/Endoplasmic Reticulum Ca^2+^-ATPase 2a; T-VGCC, T-type Voltage-Gated Calcium Channel.

Current evidence demonstrates that aberrant activation of VGCCs plays a pivotal role in sevoflurane-induced intracellular calcium overload and neurotoxicity (Zeng et al., 2022). Specifically, sevoflurane activates L-type VGCCs (L-VGCCs) in 6-days-old mice primary neurons, significantly elevating [Ca^2+^]i levels, which subsequently induces mitochondrial dysfunction and promotes neuronal apoptosis (Table 1). These effects are reversible upon application of specific L-type channel antagonists (Zeng et al., 2022). Furthermore, subanesthetic doses of sevoflurane can also activate thalamic T-type VGCCs (T-VGCCs) in aged rats, potentially representing a key mechanism underlying emergence agitation during anesthetic recovery (Shen et al., 2020; Figure 2, Loop 1). However, the study by Liu et al. (2016) unexpectedly found that sevoflurane exposure during early development stage in rats conversely inhibited L-VGCCs activity in the hippocampal region and led to neurodevelopmental impairments (Table 1). This discrepancy with previous findings may be attributed to differences in key variables including experimental models (in vitro neurons vs. in vivo systems), developmental windows (mature vs. developing neural networks), and anesthesia exposure parameters (concentration/duration). These findings not only deepen our understanding of the complexity of sevoflurane’s neuromodulatory effects but also provide important theoretical basis for optimizing clinical anesthesia protocols.

As a critical molecular target of general anesthetics, NMDAR can be specifically activated by sevoflurane through a mechanism involving sustained calcium influx caused by receptor upregulation, which subsequently triggers cascade activation of multiple apoptotic signaling pathways via calcium overload (Figure 2, Loop 2). NMDAR activation induces RIPK1 phosphorylation to initiate the necroptosis pathway (Liu et al., 2024), while concurrently activating RASD1 (Dexras1) to enhance its interaction with DMT1, thereby promoting DMT1-dependent iron uptake and lysosomal iron release, ultimately leading to ferroptosis activation (Wu et al., 2020). In addition, NMDAR activates the stress-responsive protein Ddit4 to trigger multipathway cell death programs (Li et al., 2023), while impairing synaptic plasticity in neonatal rat hippocampus and cognitive function in aged mice (Li et al., 2023; Liu et al., 2024). However, high concentrations of sevoflurane can also interfere with NMDAR membrane localization mechanisms. It significantly reduces the surface expression density of NMDAR in hippocampal neurons and inhibits activation of the NMDAR-NMNAT1/2 signaling pathway, thereby mediating neuronal inflammatory responses and cognitive dysfunction in aged rats (Tang et al., 2018; Yang et al., 2020). Other studies have also demonstrated that sevoflurane exhibits NR2A expression suppression and abnormal elevation of NR1/NR2B (Yang et al., 2020; Zhang et al., 2016), disrupting the dynamic changes in normal NMDA receptor subunit composition, which may represent a key molecular mechanism underlying its interference with neural development. Conversely, during aging processes, sevoflurane selectively reduces hippocampal NR2B subunit function, while exercise intervention can effectively reverse sevoflurane-induced cognitive impairment by restoring NR2B phosphorylation levels (Tian et al., 2016). Although these contradictory findings remain controversial, they collectively indicate that sevoflurane significantly interferes with normal NMDAR expression and function, disrupting intracellular calcium homeostasis. These discoveries further suggest that sevoflurane’s regulatory effects on NMDAR may exhibit distinct developmental stage-dependent characteristics. As the core channels regulating ER calcium release, IP3Rs and RyRs can be significantly activated by various halogenated inhaled anesthetics (including desflurane, sevoflurane, isoflurane, and halothane) (Jia et al., 2020; Osman et al., 2023; Ren et al., 2017). Sevoflurane activates IP3Rs to mediate increased [Ca^2+^]i (Pinheiro et al., 2006), with short-term effects providing cytoprotection via mTOR-dependent pathways, while prolonged exposure induces ERS, autophagic dysfunction, and hippocampal neuronal apoptosis, ultimately leading to age-related cognitive impairment (Franks et al., 1998; Liu et al., 2014; Zhang Q. et al., 2022). Sevoflurane also markedly enhances RyR1 sensitivity to Ca^2+^in both juvenile and adult rabbits (König et al., 2009), mediating intracellular calcium overload through rapid and reversible activation of RyRs. This mechanism not only causes necrosis in isolated rat hippocampal neurons (Zhang Q. et al., 2022) but is also considered a key molecular basis for inhaled anesthetic-related malignant hyperthermia (Figure 2, Loop 3).

Simultaneously, sevoflurane differentially modulates myocardial calcium handling through the SERCA2a/PLB system. In a rat model of pulmonary hypertension, it downregulates SERCA2a expression while upregulating its inhibitory protein PLB (phospholamban), resulting in reduced sarcoplasmic reticulum calcium uptake capacity and impaired left ventricular contractile function (Wang et al., 2017; Figure 2, Loop 4). Conversely, it enhances sarcoplasmic reticulum calcium uptake in adult rabbit models (Laver et al., 2017). *In vitro* experiments demonstrate that SERCA inhibitors partially attenuate sevoflurane-induced intracellular calcium elevation (Liu et al., 2024). SERCA activity is regulated by hydroxyl radicals and PLB phosphorylation status, and sevoflurane may alter SERCA function by increasing cellular H_2_O_2_ production (a critical hydroxyl radical precursor) (Dixon et al., 2012) and upregulating PKA (the PLB kinase) (Liu T. J. et al., 2018; Figure 2, Loop 5).

As a calcium ion-dependent protease, calpain’s enzymatic activity is strictly regulated by [Ca^2+^]c under physiological conditions. The activity of the calpain system significantly increases during aging (Uryash et al., 2024), and in its activated state, it can cleave and activate downstream target proteins (such as apoptosis-related factors and inflammatory mediators), regulating various physiological and pathological processes including apoptosis and inflammatory responses (Han et al., 2018). In addition to indirectly activating calpain by disrupting [Ca^2+^]c, sevoflurane exposure directly enhances calpain expression and activity through non-calcium-dependent pathways, including microRNA-mediated post-transcriptional regulation (e.g., downregulation of miR-124) and protein-protein interactions (e.g., CD44-calpain complex formation), leading to cascading hippocampal neuronal apoptosis, neuroinflammatory responses in aged rats (Table 1) (Zhao Z. et al., 2021), and glioblastoma cell invasion (Lai et al., 2019). This may represent one of the key molecular mechanisms underlying sevoflurane-induced neurotoxicity (Figure 2, Loop 6). Elevated [Ca^2+^]c disrupts ER calcium homeostasis and induces mitochondrial membrane potential (ΔΨm) collapse, while concurrently activating calmodulin (CaM)-dependent signaling pathways and calpain-mediated proteolysis of target proteins. Sevoflurane interferes with SERCA pump function, ryanodine receptor (RyR) activity, and voltage-gated calcium channel regulation, while modulating calpain activation, collectively disrupting neuronal calcium ion homeostasis and inducing neurotoxicity. Future research should prioritize the development of calcium homeostasis-targeted therapeutic strategies to mitigate sevoflurane-induced neurotoxicity and enhance clinical safety.

### 2.3 BDNF

**FIGURE 3:**
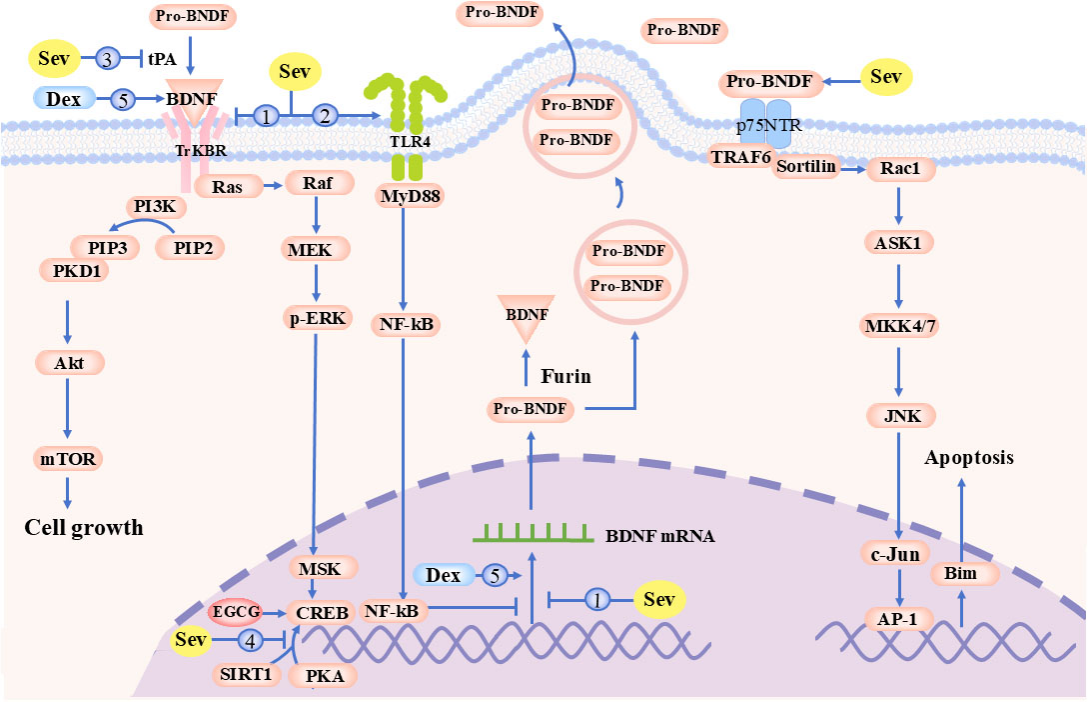
Mechanisms of Sevoflurane-Induced BDNF Impairment. AP-1, Activator Protein 1; Akt, Protein Kinase B (PKB); ASK1, Apoptosis Signal-regulating Kinase 1; BDNF, Brain-Derived Neurotrophic Factor; Bim, Bcl-2 Interacting Mediator of cell death; c-Jun, Jun Proto-Oncogene; CREB, cAMP Response Element-Binding Protein; Dex, Dexamethasone; EGCG, Epigallocatechin Gallate; JNK, c-Jun N-terminal Kinase; MEK, Mitogen Activated Protein Kinase Kinase; MKK4/7, Mitogen-Activated Protein Kinase Kinases 4/7; mTOR, Mechanistic Target of Rapamycin; MSK, Mitogen- and Stress-activated Kinase; NF-kB, Nuclear Factor Kappa light chain enhancer of Activated B cells; p75NTR, p75 Neurotrophin Receptor; PI3K, Phosphoinositide 3-Kinase; PIP2, Phosphatidylinositol 4,5-Bisphosphate; PKA, Protein Kinase A; PKD1, Protein Kinase D1; Rac1, Ras-related C3 Botulinum Toxin Substrate 1; Raf, Rapidly Accelerated Fibrosarcoma Kinase; Ras, Rat Sarcoma Viral Oncogene Homolog; SIRT1, Sirtuin 1; TRAF6, TNF Receptor-Associated Factor 6; TrkBR, Tropomyosin Receptor Kinase B; tPA, Tissue Plasminogen Activator.

As a crucial member of the neurotrophic factor family, BDNF plays a central role in neural development and functional maintenance by binding to tyrosine kinase receptor B (TrkB) to activate two key signaling pathways: (1) the PI3K/Akt/mTOR pathway promoting synaptic plasticity-related protein synthesis and dendritic spine morphogenesis (Xu and Qian, 2020); (2) the ERK/CREB pathway forming a positive feedback regulatory loop to enhance BDNF autotranscription (Dong et al., 2020a), thereby coordinately regulating advanced neural functions including hippocampus-dependent learning and memory, emotional responses, and anxiety-like behaviors. Current research has established the BDNF/TrkB signaling system as a core molecular target in sevoflurane-induced cognitive impairment, with sevoflurane disrupting BDNF’s neuroprotective effects through three mechanisms: (1) direct inhibition of BDNF and TrkB gene transcription in juvenile rats (Dong et al., 2020a; Figure 3, Loop 1); (2) blockade of the BDNF/TrkB/CREB signaling cascade in offspring rats via TLR4 upregulation (Li Q. Q. et al., 2025; Figure 3, Loop 2); and (3) suppression of proBDNF-to-mBDNF conversion in developing rats by interfering with the tPA/PAI-1 protease system, leading to abnormal activation of proBDNF-mediated apoptotic pathways and subsequent synaptic structural damage (Table 1) (Dong et al., 2020a; Dong et al., 2020b; Figure 3, Loop 3). Notably, intervention studies employing hippocampal BDNF overexpression or TrkB pathway-specific activation have successfully reversed sevoflurane-induced neurotoxicity in aged rats (Li Q. Q. et al., 2025; Yin et al., 2022), not only validating the central role of this pathway but also identifying potential therapeutic targets for clinical prevention and treatment.

Recent studies have revealed that sevoflurane can regulate BDNF expression through epigenetic mechanisms: repeated exposure during developmental stages significantly increases hippocampal histone deacetylation levels in neonatal rats, thereby suppressing BDNF transcription and leading to neurodevelopmental deficits (Jia et al., 2016); this effect can be effectively reversed by histone deacetylase inhibitors (such as entinostat and sodium butyrate) (Jia et al., 2016; Joksimovic et al., 2018). Furthermore, sevoflurane induces hypermethylation of the BDNF gene promoter region, not only reducing BDNF expression levels but also causing a significant decrease in dendritic spine density of pyramidal neurons in the hippocampal CA1 area (Ju et al., 2016), further confirming the critical role of epigenetic modifications in sevoflurane-induced neurotoxicity in neonatal rats. In addition, the promoter region of the BDNF gene contains several CREB-binding sites, and CREB phosphorylation is essential for BDNF expression and function. Current studies have demonstrated that sevoflurane inactivates CREB, a key epigenetic regulator of BDNF, by suppressing SIRT1, PKA, and ovarian cancer G-protein-coupled receptor 1 (GPR68), thereby reducing hippocampal BDNF expression and mediating cognitive dysfunction and learning/memory impairments in experimental animals (Tang X. et al., 2020; Zhao J. et al., 2021; Zhao et al., 2022; Figure 3, Loop 4). Wang’s study further revealed that sevoflurane anesthesia upregulates the expression of long non-coding RNA HOTAIR, promoting its binding to the transcriptional repressor complex Sin3A/coREST and enhancing RE-1 silencing transcription factor (REST)-mediated suppression of the BDNF gene, ultimately inducing cognitive dysfunction-like manifestations in aged rats (Table 1) (Wang et al., 2018). Notably, targeted interventions in these pathways [such as activating SIRT1/PKA (Tang X. et al., 2020; Zhao J. et al., 2021), overexpressing GPR68 (Zhao et al., 2022), or inhibiting HOTAIR (Wang et al., 2018)] can effectively reverse sevoflurane-induced BDNF expression suppression and cognitive impairment, providing potential therapeutic targets for clinical prevention and treatment.

Brain-derived neurotrophic factor, as a critical neurotrophic factor, activates TrkB downstream PI3K/Akt and MAPK/ERK pathways to exert anti-apoptotic, pro-synaptic growth, and neuroprotective effects. Consequently, enhancing BDNF expression (e.g., through pharmacological interventions, exercise, or environmental enrichment) or directly targeting the BDNF/TrkB pathway has progressively emerged as an effective strategy for preventing or treating sevoflurane-induced neurotoxicity, providing new directions for perioperative neuroprotection. The selective α2-adrenergic receptor agonist dexmedetomidine (DEX) upregulates hippocampal BDNF and its receptors, alleviating sevoflurane-induced neurotoxicity in late-pregnancy rats (Dong et al., 2020a; Figure 3, Loop 5). Elevated plasma BDNF levels and TrkB activation status in young rats can ameliorate sevoflurane-induced hippocampal synaptic plasticity impairment and cognitive dysfunction (Li Y. et al., 2022). Epigallocatechin-3-gallate (EGCG) activates the CREB/BDNF/TrkB-PI3K/Akt signaling pathway, effectively inhibiting sevoflurane-induced neurodegeneration in mice and improving learning and memory performance (Ding et al., 2017). Meanwhile, the tPA and PAI-1 inhibitor TM5275 blocks upregulated proBDNF and PAI-1 protein expression in rat hippocampus, increases BDNF and TrkB activity, and reverses sevoflurane-induced learning/memory deficits and reduced hippocampal synaptic plasticity (Dong et al., 2020b).

### 2.4 Inflammatory mechanisms

Inflammation can directly or indirectly impair learning and memory functions by interfering with intracellular signaling pathways in neurons and exacerbate neuronal damage induced by sevoflurane anesthesia, thereby promoting the occurrence of developmental neurotoxicity (Useinovic et al., 2022). Simultaneously, sevoflurane anesthesia may also activate inflammation-related signaling pathways, leading to the excessive production of pro-inflammatory cytokines (such as TNF-α, IL-1β, and IL-6) in the brain, further aggravating neuroinflammatory responses and ultimately resulting in age-related cognitive dysfunction (Cui et al., 2018). This bidirectional vicious cycle mechanism is likely the key pathological basis of sevoflurane-induced neurotoxicity. In recent years, with the continuous advancement of molecular biology and neuroimmunology research, various inflammation-regulating mechanisms mediated by sevoflurane have been gradually elucidated.

#### 2.4.1 Microglial activation

Microglia are specialized resident immune cells in the central nervous system that continuously sample the microenvironment through their highly dynamic processes, performing immune surveillance functions. Studies indicate that sevoflurane promotes M1 (pro-inflammatory) polarization and suppresses M2 (anti-inflammatory) polarization of microglia by inhibiting the Maf1/AMPK pathway and increasing Triggering Receptor Expressed on Myeloid Cells-1 (TREM1) expression (Tang et al., 2023), thereby mediating inflammatory damage (Dai et al., 2022). Additionally, in aged mice, sevoflurane downregulates isatin and PPARγ, releasing their inhibitory effects on hippocampal microglia, and induces proliferation of hippocampal astrocytes and microglia, accompanied by elevated expression of inflammatory factors (Huang and Zhu, 2024; Wang T. et al., 2024). On the other hand, sevoflurane removes inhibitory neuronal signals through endogenous damage-associated molecular patterns (DAMPs) and activates microglia by engaging pattern recognition receptors. It significantly promotes the release of endogenous danger signal molecules–high mobility group box 1 (HMGB-1) and galectin-3–in hippocampal tissue. These molecules specifically activate microglial surface pattern recognition receptors TLR4 and TLR9, triggering the classical MyD88-dependent signaling pathway and activating PI3K/Akt. This leads to a marked increase in the phosphorylation level of IκBα, a key regulatory factor of NF-κB, thereby enhancing the transcription and release of downstream pro-inflammatory cytokines, including TNF-α, IL-1β, and IL-6. Delaying the release of HMGB-1 and galectin-3 (Joe et al., 2024) or knocking out TLR4 in mice (Xiang et al., 2020) can reverse the neuroinflammatory effects induced by sevoflurane, providing potential intervention targets for the clinical prevention and treatment of anesthesia-related cognitive dysfunction. However, in sepsis models, sevoflurane interferes with the formation of the TLR4-MD-2-LPS complex in a dose-dependent manner, inhibits TLR4 activation, attenuates NF-κB signaling, and exerts anti-inflammatory effects (Okuno et al., 2019). Therefore, sevoflurane may have both protective and detrimental roles in neuroinflammation, with its impact on inflammatory responses varying across species and underlying disease conditions.

#### 2.4.2 Downregulation of the cholinergic anti-inflammatory pathway

The α7 nicotinic acetylcholine receptor (α7nAChR) is expressed on both non-neuronal and neuronal cells and is activated upon binding acetylcholine (ACh). This activation suppresses nuclear translocation of NF-κB in macrophages, reducing the release of pro-inflammatory cytokines (e.g., TNF-α, IL-1β, IL-6), while simultaneously activating the JAK2-STAT3 pathway to enhance the expression of anti-inflammatory factors (e.g., IL-10) (Wang et al., 2003). Repeated exposure to clinical concentrations of sevoflurane, on one hand, suppresses acetylcholinesterase expression in the rat hippocampus, increasing ACh degradation (Yin et al., 2019), and on the other hand, reduces presynaptic ACh release by inhibiting calcium currents (Pavlov and Tracey, 2005), thereby preventing α7nAChR activation. Sevoflurane also elevates TNF-α levels in plasma and hippocampal tissues, impairing cognitive function in rats (Yin et al., 2019). Notably, the α7nAChR agonist PNU-282987 can reverse this toxic effect (Yin et al., 2019), further confirming that inflammation in sevoflurane-induced cognitive decline is associated with the downregulation of the α7nAChR-mediated cholinergic anti-inflammatory pathway in aged rats.

#### 2.4.3 NLRP1 inflammasome activation

The NLRP1 inflammasome-driven inflammatory response has been implicated in numerous neurological disorders. As the first member of the NLR family to form an inflammasome complex and activate caspase-1, NLRP1 triggers both inflammatory responses and pyroptosis through the activation of IL-1β and Gasdermin D (GSDMD) (Gong et al., 2021). Inhibition of NLRP1/caspase-1 signaling has been shown to confer neuroprotection in Alzheimer’s disease models (Yap et al., 2019). *In vivo* and *in vitro* studies confirm that sevoflurane exposure upregulates hippocampal expression of NLRP1, cleaved caspase-1, GSDMD-N, IL-1β, and IL-18 in neonatal rats (Table 1) (Useinovic et al., 2022; Wang Z. et al., 2024). Mechanistically, in neonatal mice, sevoflurane suppresses SET domain-containing 1B (SETD1B), reducing H3K4me3 levels at the CXCR4 promoter and thereby downregulating CXCR4 expression. This relieves its inhibitory effect on the NLRP1/caspase-1 pathway, ultimately promoting hippocampal neuronal pyroptosis (Wang Z. et al., 2024). Additionally, studies demonstrate that sevoflurane epigenetically upregulates the long non-coding RNA H19 in the neonatal mouse hippocampus, increasing the expression of the deubiquitinase USP30. This suppresses damaged mitophagy, leading to mitochondrial dysfunction, ROS accumulation, and subsequent NLRP1 inflammasome assembly and caspase-1 activation in microglia. The resulting overproduction of inflammatory cytokines (e.g., IL-1β, IL-18) drives neuronal pyroptosis, contributing to spatial memory deficits and emotional cognitive impairment (Gao and Huang, 2024).

Accumulating preclinical evidence has established the central role of neuroinflammatory mechanisms in sevoflurane-induced neurotoxicity, particularly the aberrant activation of the TLR4/NF-κB signaling pathway and the polarization of microglia toward the pro-inflammatory M1 phenotype. These findings not only elucidate the molecular pathogenesis of sevoflurane neurotoxicity but, more importantly, provide a theoretical foundation for developing targeted anti-inflammatory therapeutic strategies. Notably, researchers have successfully mitigated sevoflurane-induced neuronal damage by specifically inhibiting these inflammatory signaling pathways, demonstrating promising clinical translation potential. Intravenous administration of IGF-1 in rats restored sevoflurane-suppressed Akt phosphorylation, modulated the PI3K/Akt signaling pathway, and alleviated sevoflurane-induced cognitive impairment in aged rats (Xie et al., 2021). Specific knockout of the hippocampal inflammatory key regulator TREM1 in aged mice reduced microglial M1 polarization, ameliorating neuroinflammation and sevoflurane-induced cognitive dysfunction (Tang et al., 2023). miR-424 mitigated the adverse effects of sevoflurane by specifically recognizing and binding to the 3′- UTR of TLR4, promoting its mRNA degradation (Li Z. et al., 2022). Meanwhile, the traditional Chinese medicine Morroniside not only promoted microglial M2 polarization but also bound to TLR4 to block TLR4/NF-κB pathway activation, collectively alleviating Sev-induced hippocampal tissue damage and neuroinflammation, and it was the first to improve cognitive dysfunction in aged mice (Chen et al., 2024). Targeting the regulation of neuroinflammatory responses (e.g., using TLR4 inhibitors or pro-M2 polarization drugs) is expected to significantly reduce the neurocognitive side effects of sevoflurane while maintaining anesthetic efficacy. This approach is particularly important for brain protection in high-risk populations such as elderly and pediatric patients.

### 2.5 ERS

In the 1980s, researchers first discovered that ER dysfunction under cellular stress or environmental disturbances leads to the accumulation of unfolded/misfolded proteins, thereby activating three classical unfolded protein response (UPR) pathways mediated by IRE1α, PERK, and ATF6α (Kozutsumi et al., 1988). If cells can rapidly alleviate the accumulation of unfolded proteins, they effectively adapt to and mitigate resulting cellular damage. However, when persistent stress exceeds the organism’s adaptive capacity, the UPR transitions from a protective mechanism to a pro-apoptotic signaling pathway. This shift may ultimately trigger programmed cell death, eliminating damaged cells to maintain the organism’s overall homeostasis.

In recent years, a growing body of research has attributed neurological disorders induced by inhaled anesthetics to widespread activation of ERS, particularly more pronounced in the developing and aging brain. Wang et al. found that repeated sevoflurane exposure in aged mice resulted in significant ER dilation in the hippocampal region, accompanied by activation of the PERK/eIF2α/ATF4 pathway and upregulation of the molecular chaperone GRP78 expression, suggesting that sevoflurane may mediate neurotoxicity by inducing ERS (Wang Y. et al., 2023). Notably, in animal studies, the pathological effects of this ERS exhibited significant hippocampal subregion heterogeneity–ERS in CA1 pyramidal neurons ultimately led to calcium channel dysfunction and weakened neuronal intrinsic activity, while dentate gyrus neural stem cells exhibited caspase 12-mediated apoptosis (Zhu et al., 2017). Mechanistically, activation of the PERK/eIF2α/ATF4 pathway, on one hand, reduced CREB1 and BDNF levels while upregulating CHOP expression, inhibited the anti-apoptotic protein Bcl-2, and activated the pro-apoptotic protein Bax, thereby promoting hippocampal cell apoptosis and cognitive dysfunction in aged mice (Table 1) (Wang Y. et al., 2023). On the other hand, ATF4 overexpression can further induce transcriptional activation and nuclear translocation of ATF3, leading to abnormal intracellular H_2_O_2_ accumulation through upregulation of key oxidative stress effector molecules (e.g., NOX4), ultimately promoting neuronal ferroptosis and spatial memory dysfunction in developing mice exposed to sevoflurane (Liu et al., 2017). Beyond the hippocampus, sevoflurane-induced ERS has also been observed in both aged and neonatal mouse cerebral cortex tissues. Sevoflurane activates the cortical PERK-eIF2α-ATF4-CHOP signaling pathway, inducing apoptosis in neonatal mouse cortical neurons while causing neuronal hyperexcitability, synaptic loss, and cognitive decline in the frontal cortex of aged mice, with PERK inhibitors effectively mitigating these effects (Chen K. et al., 2022; Liu et al., 2017).

The mechanism by which sevoflurane induces ERS and activates the PERK-eIF2α-ATF4 signaling pathway has become a current research focus, with existing evidence suggesting this process may involve multiple synergistic mechanisms: (1) upregulation of phosphorylated IP3R expression, disrupting intracellular calcium ion homeostasis (Zhang Q. et al., 2022); (2) promoting miR-15b-5p overexpression, specifically targeting and suppressing the key ERS regulator Rab1A (Li et al., 2017); (3) directly activating the downstream PERK signaling pathway through upregulation of protein tyrosine phosphatase 1B (PTP1B) (Liu et al., 2019). These findings indicate that the PERK signaling pathway may serve as a critical regulatory target in sevoflurane-induced neurodegeneration, not only providing novel theoretical perspectives for elucidating the molecular mechanisms of sevoflurane neurotoxicity but also opening potential therapeutic strategies for clinically preventing its neurotoxic side effects.

Notably, multiple disease model studies have demonstrated that sevoflurane can exert significant protective effects by effectively inhibiting ERS. It suppresses the PLCγ/CaMKII/IP3R signaling pathway in neuropathic pain models, alleviating ERS and inflammatory responses in rats (Xie et al., 2024). By inhibiting the IRE1-mediated endoplasmic reticulum stress pathway, it mitigates hypoxic-ischemic brain injury, hemorrhagic shock, and lipopolysaccharide-induced apoptosis in human umbilical vein endothelial cells (Hu et al., 2018; Ni W. et al., 2024; Niu et al., 2021). Furthermore, through suppression of the PERK-eIF2α pathway, it ameliorates ischemia-reperfusion injury in peripheral organs and cellular oxidative stress damage in rats (Cuan et al., 2024; Liu D. et al., 2018). Sevoflurane’s regulation of ERS exhibits distinct concentration-dependent, cell-type-specific, and pathology-selective characteristics. Building on these findings, researchers have begun developing intervention strategies targeting key ERS molecules, achieving significant progress in preclinical studies: for instance, the eIF2α dephosphorylation inhibitor Salubrinal and PERK inhibitor GSK2606414 have effectively improved cognitive dysfunction in sevoflurane-exposed mice (Chen K. et al., 2022; Wang Y. et al., 2023). However, current evidence remains largely confined to animal models, and clinical translation faces major challenges including drug specificity, long-term safety, and human pharmacodynamic validation. Future research requires multicenter clinical trials and translational medicine studies to further evaluate the feasibility and efficacy of these intervention strategies in clinical applications.

## 3 Evidence-based strategies and challenges in mitigating sevoflurane neurotoxicity: focus on DEX and melatonin

Growing preclinical and clinical evidence has established DEX and melatonin as promising pharmacological interventions against sevoflurane-induced neurotoxicity. These agents exhibit distinct yet complementary neuroprotective mechanisms, targeting multiple pathways involved in neuronal damage, inflammation, and oxidative stress associated with sevoflurane exposure across different age groups.

### 3.1 DEX: a multi-target neuroprotectant

As a selective α2-adrenoceptor agonist, DEX demonstrates comprehensive neuroprotective effects through multiple molecular pathways. It modulates BDNF-TrkB-CREB/ProBDNF-P75NRT-RhoA signaling to preserve neuronal development and cognitive function in late-pregnancy rats (Dong et al., 2020a; Zeng et al., 2022), activates BMP/SMAD pathway to maintain cytoskeletal integrity and reduce apoptosis in offspring rats (Shan et al., 2018), and suppresses miR-204-5p/SOX4 and miR-330-3p/ULK1 axes to mitigate oxidative stress and impaired mitophagy in rat hippocampus (Wang R. et al., 2023; Xu S. et al., 2021). Clinically, DEX decreases inflammatory cytokines in elderly patients, reducing POCD incidence, while in pediatric populations it effectively reduces emergence agitation and prevents postoperative behavioral changes (Shi et al., 2019; Tang W. et al., 2020; Zhang et al., 2018).

### 3.2 Melatonin: a multimodal therapeutic agent

Melatonin, as an endogenous indoleamine, exhibits pleiotropic properties by enhancing mitophagy to suppress microglial activation and improve cognitive deficits in neonatal rodents (Zhang et al., 2023), modulating Wnt/β-catenin signaling via MT1 receptors to ameliorate synaptic toxicity (Liang et al., 2021), and demonstrating age-specific protection mechanisms - regulating apoptosis and inflammation in preadolescent rats (Heydari et al., 2024) while activating Nrf2 pathway and downregulating PI3K/Akt/mTOR in aged mice (Ni H. et al., 2024; Shen et al., 2022). Clinically, it serves as an effective preoperative medication for children, reducing both anxiety and postoperative delirium (Jangra et al., 2022).

Although melatonin and DEX have demonstrated neuroprotective effects in animal models, their clinical translation faces significant challenges due to poor blood-brain barrier (BBB) penetration, off-target effects, and dose-dependent paradoxical effects (Chuffa et al., 2021; Fatouh et al., 2017). However, emerging technologies offer promising solutions to these limitations. When combined with nanoformulations, both melatonin and DEX exhibit prolonged release profiles, enhanced efficacy, and improved safety (Chen Y. L. et al., 2022; Chuffa et al., 2021). For instance, the melatonin receptor agonist agomelatine, when loaded into nanoemulsions and administered intranasally, shows significantly increased brain bioavailability (Fatouh et al., 2017). Similarly, DEX-loaded gold nanoparticles (AuNPs-DEX) effectively mitigate neurocognitive impairment in anesthetized rats (Zhang et al., 2021).

Furthermore, given that sevoflurane-induced neurotoxicity is often associated with miRNA dysregulation, exosome-mediated delivery of therapeutic RNAs to target cells has emerged as a potential strategy to correct protein dysfunction and alleviate post-anesthetic neurotoxicity. Exosomes derived from mesenchymal stem cells (MSCs) have shown the ability to repair neuronal damage and reduce the neurotoxicity of anesthetics (Gu and Zhu, 2021). Additionally, cerebrospinal fluid (CSF)-derived exosomes can promote the proliferation of neuronal cells *in vitro*, thereby contributing to neuronal repair processes (Kong et al., 2018).

These evidence-based strategies–including DEX targeting anti-inflammatory pathways and melatonin employing multimodal mechanisms–offer promising avenues to mitigate sevoflurane-induced neurotoxicity. The integration of nanotechnology (e.g., nanoparticle formulations) and exosome-based delivery systems further enhances their potential by overcoming pharmacokinetic limitations.

## 4 Summary and future perspectives

**FIGURE 4:**
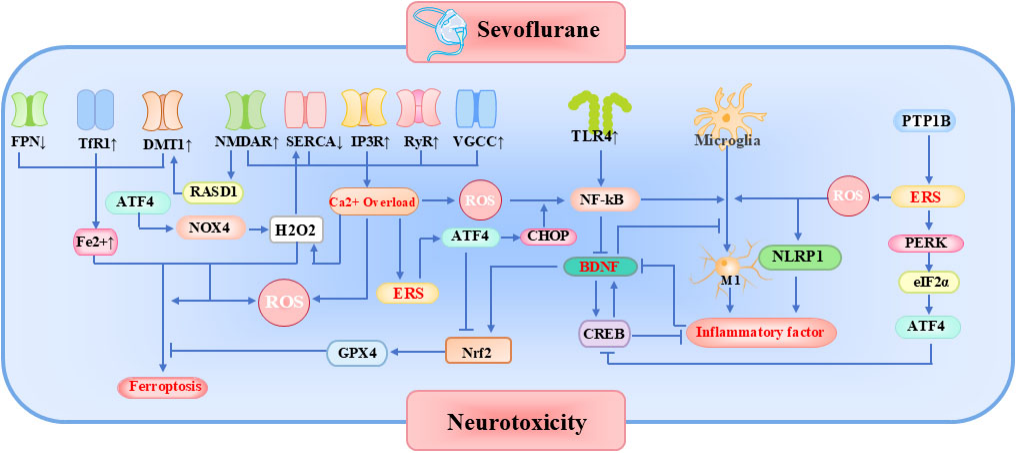
Mechanisms of Sevoflurane Neurotoxicity. Mechanisms highlighted in red represent the five key mechanisms examined in this study. ATF4, Activating Transcription Factor 4; BDNF, Brain-Derived Neurotrophic Factor; CHOP, C/EBP homologous protein; CREB, cAMP Response Element-Binding Protein; DMT1, Divalent Metal Transporter 1; eIF2α, Eukaryotic Initiation Factor 2 Subunit Alpha; ERS, Endoplasmic Reticulum Stress; FPN, Ferroportin; GPX4, Glutathione Peroxidase 4; IP3R, Inositol 1,4,5-Trisphosphate Receptor; NF-Kb, Nuclear Factor Kappa B; NLRP1, NLR Family Pyrin Domain Containing 1; NMDAR, N-Methyl-D-Aspartate Receptor; NOX4, NADPH Oxidase 4; Nrf2, Nuclear factor erythroid 2-related factor 2; PERK, Protein Kinase R-like Endoplasmic Reticulum Kinase; PTP1B, Protein Tyrosine Phosphatase 1B; RASD1, RAS Dexamethasone-Induced 1; ROS, Reactive Oxygen Species; RyR, Ryanodine Receptor; SERCA, Sarco/Endoplasmic Reticulum Ca^2+^ ATPase; TLR4, Toll-Like Receptor 4; TfR1, Transferrin Receptor 1; VGCC, Voltage-Gated Calcium Channel.

With advancements in molecular biology and neuroscience technologies, the multifaceted mechanisms underlying sevoflurane-induced neurotoxicity have been increasingly delineated, revealing interconnected dysregulation in endoplasmic reticulum stress, ferroptosis activation, BDNF/TrkB signaling pathways, and neuroinflammatory cascades as illustrated in Figure 4. These pathophysiological processes collectively contribute to neural damage through synergistic or antagonistic interactions, establishing a theoretical foundation for targeted therapeutic interventions. Nevertheless, a significant translational gap persists between experimentally identified molecular mechanisms and clinical efficacy, primarily due to species-specific neurodevelopmental disparities between animal models and humans, pharmacokinetic discrepancies between *in vitro* concentrations and clinical dosing regimens, and methodological limitations in correlating short-term observational biomarkers with long-term neurocognitive outcomes. Consequently, direct clinical translation of current fundamental research findings remains substantially constrained, necessitating future prioritization of translational medicine approaches and prospective large-scale clinical trials to validate mechanistic insights and develop etiology-specific neuroprotective strategies.

## References

[B1] Ay B. Wallace D. Mantilla C. B. Prakash Y. S. (2005). Differential inhibition of neuronal Na+-Ca2+ exchange versus store-operated Ca2+ channels by volatile anesthetics in pheochromocytoma (PC12) cells. *Anesthesiology* 103 93–101. 10.1097/00000542-200507000-00016 15983461

[B2] Chen J. Peng B. Lin W. Mao Y. Wang Y. (2024). Morroniside ameliorates sevoflurane anesthesia-induced cognitive dysfunction in aged mice through modulating the TLR4/NF-κB pathway. *Biomol. Biomed.* 10.17305/bb.2024.11433 Online ahead of print. 39670511 PMC12447737

[B3] Chen K. Hu Q. Gupta R. Stephens J. Xie Z. Yang G. (2022). Inhibition of unfolded protein response prevents post-anesthesia neuronal hyperactivity and synapse loss in aged mice. *Aging Cell* 21:e13592. 10.1111/acel.13592 35299279 PMC9009124

[B4] Chen X. Zhao M. White P. F. Li S. Tang J. Wender R. H. (2001). The recovery of cognitive function after general anesthesia in elderly patients: A comparison of desflurane and sevoflurane. *Anesthesia Analgesia* 93 1489–1494. 10.1097/00000539-200112000-00029 11726429

[B5] Chen Y. L. Lai J. Y. Zhou R. F. Ouyang T. F. Fu H. (2022). The analgesic effect of dexmedetomidine loaded with nano-hydrogel as a novel nano-drug delivery system for thoracic paravertebral block after thoracic surgery. *J. Biomed. Nanotechnol.* 18 1604–1612. 10.1166/jbn.2022.3377

[B6] Chuffa L. G. Seiva F. R. F. Novais A. A. Simao V. A. Giménez V. M. M. Manucha W. (2021). Melatonin-loaded nanocarriers: New horizons for therapeutic applications. *Molecules* 26:3562. 10.3390/molecules26123562 34200947 PMC8230720

[B7] ClinicalTrials.gov. (2020). *Sevoflurane, propofol and desflurane on POD/POCD. ClinicalTrials.gov identifier: NCT03326960.* Available online at: https://clinicaltrials.gov/study/NCT03326960?cond=sevoflurane&rank=13 (accessed October 22, 2022).

[B8] ClinicalTrials.gov. (2024). *Sevoflurane General anesthetic and spatial memory in humans. ClinicalTrials.gov identifier: NCT05991817.* Available online at: https://clinicaltrials.gov/study/NCT05991817?cond=sevoflurane&page=5&rank=114 (accessed January 26, 2024).

[B9] Cuan J. Wu C. Zheng F. Lu X. Waqas M. U. (2024). Sevoflurane pretreatment enhances myocardial ischemia-reperfusion injury via activating HIF-1α/iNOS/cGMP to inhibit endoplasmic reticulum stress. *Pak. Vet. J.* 44, 785–793. 10.29261/pakvetj/2024.257 40121575

[B10] Cui R. S. Wang K. Wang Z. L. (2018). Sevoflurane anesthesia alters cognitive function by activating inflammation and cell death in rats. *Exp. Therapeutic Med.* 15 4127–4130. 10.3892/etm.2018.5976 29849771 PMC5962843

[B11] Dai Y. Yan M. Wan J. Xiao T. (2022). Maf1 mitigates sevoflurane-induced microglial inflammatory damage and attenuates microglia-mediated neurotoxicity in HT-22 cells by activating the AMPK/Nrf2 signaling. *NeuroToxicology* 90 237–245. 10.1016/j.neuro.2022.04.003 35430185

[B12] DiMaggio C. Sun L. S. Li G. (2011). Early childhood exposure to anesthesia and risk of developmental and behavioral disorders in a sibling birth cohort. *Anesthesia Analgesia* 113 1143–1151. 10.1213/ANE.0b013e3182147f42 21415431 PMC3164160

[B13] Ding M. L. Ma H. Man G. Lv Y. (2017). Protective effects of a green tea polyphenol, epigallocatechin-3-gallate, against sevoflurane-induced neuronal apoptosis involve regulation of CREB/BDNF/TrkB and PI3K/Akt/mTOR signalling pathways in neonatal mice. *Can. J. Physiol. Pharmacol.* 95 1396–1405. 10.1139/cjpp-2016-0333 28679060

[B14] Dixon S. J. Lemberg K. M. Lamprecht M. R. Skouta R. Zaitsev E. M. Gleason C. E. (2012). Ferroptosis: An iron-dependent form of nonapoptotic cell death. *Cell* 149 1060–1072. 10.1016/j.cell.2012.03.042 22632970 PMC3367386

[B15] Dong Y. Hong W. Tang Z. Gao Y. Wu X. Liu H. (2020a). Dexmedetomidine attenuates neurotoxicity in developing rats induced by sevoflurane through upregulating BDNF-TrkB-CREB and downregulating ProBDNF-P75NRT-RhoA signaling pathway. *Mediators Inflammation* 2020:5458061. 10.1155/2020/5458061 32655312 PMC7322616

[B16] Dong Y. Hong W. Tang Z. Gao Y. Wu X. Liu H. (2020b). Sevoflurane leads to learning and memory dysfunction via breaking the balance of tPA/PAI-1. *Neurochem. Int.* 139:104789. 10.1016/j.neuint.2020.104789 32650025

[B17] Fang F. Song R. Ling X. Peng M. Xue Z. Cang J. (2017). Multiple sevoflurane anesthesia in pregnant mice inhibits neurogenesis of fetal hippocampus via repressing transcription factor Pax6. *Life Sci.* 175 16–22. 10.1016/j.lfs.2017.03.003 28279665

[B18] Fatouh A. M. Elshafeey A. H. Abdelbary A. (2017). Intranasal agomelatine solid lipid nanoparticles to enhance brain delivery: Formulation, optimization and in vivo pharmacokinetics. *Drug Design Development Therapy* 11 1815–1825. 10.2147/DDDT.S102500 28684900 PMC5484509

[B19] Franks J. J. Wamil A. W. Janicki P. K. Horn J. L. Franks W. T. Janson V. E. (1998). Anesthetic-induced alteration of Ca2+ homeostasis in neural cells: A temperature-sensitive process that is enhanced by blockade of plasma membrane Ca2+-ATPase isoforms. *Anesthesiology* 89 149–164. 10.1097/00000542-199807000-00022 9667305

[B20] Gao T. Huang Z. (2024). Novel insights into sevoflurane-induced developmental neurotoxicity mechanisms. *Epigenomics* 16 1231–1252. 10.1080/17501911.2024.2395250 39316776 PMC11485883

[B21] Gomes D. A. Guatimosim C. Gomez R. S. Leite M. F. Vieira L. B. Prado M. A. (2004). Effect of halothane on the release of [Ca2+]i in dorsal root ganglion neurons. *NeuroReport* 15 1187–1190. 10.1097/00001756-200405190-00021 15129171

[B22] Gong H. Wan X. Zhang Y. Liang S. (2021). Downregulation of HOTAIR reduces neuronal pyroptosis by targeting miR-455-3p/NLRP1 axis in propofol-treated neurons in vitro. *Neurochem. Res.* 46 1141–1150. 10.1007/s11064-021-03249-6 33534059

[B23] Gu X. Zhu J. (2021). Roles of exosomes and exosomal MicroRNAs in postoperative sleep disturbance. *Nat. Sci. Sleep* 13 1363–1375. 10.2147/NSS.S310351 34354381 PMC8331078

[B24] Han X. Liu C. Zhang K. Guo M. Shen Z. Liu Y. (2018). Calpain and JNK pathways participate in isoflurane-induced nucleus translocation of apoptosis-inducing factor in the brain of neonatal rats. *Toxicol. Lett.* 285 60–73. 10.1016/j.toxlet.2017.12.022 29289695

[B25] He K. Li Y. Xiong W. Xing Y. Gao W. Du Y. (2024). Sevoflurane exposure accelerates the onset of cognitive impairment via promoting p-Drp1S616-mediated mitochondrial fission in a mouse model of Alzheimer’s disease. *Free Radical Biol. Med.* 225 699–710. 10.1016/j.freeradbiomed.2024.10.301 39490772

[B26] Heydari F. Nasiri M. Haroabadi A. Fahanik Babaei J. Pestehei S. K. (2024). Efficacy of melatonin in alleviating disorders arising from repeated exposure to sevoflurane in males and females of the Wistar rats during preadolescence. *Sci. Rep.* 14:11889. 10.1038/s41598-024-62170-4 38789558 PMC11126601

[B27] Hu X. Zhang M. Duan X. Zhang Q. Huang C. Huang L. (2018). Sevoflurane postconditioning improves the spatial learning and memory impairments induced by hemorrhagic shock and resuscitation through suppressing IRE1α-Caspase-12-mediated endoplasmic reticulum stress pathway. *Neurosci. Lett.* 685 160–166. 10.1016/j.neulet.2018.08.035 30157449

[B28] Hu X. Zhang Y. Guo L. Xiao R. Yuan L. Liu F. (2024). Comprehensive Analysis of bulk RNA-Seq and single-cell RNA-seq data unveils sevoflurane-induced neurotoxicity through SLC7A11-associated ferroptosis. *J. Cell. Mol. Med.* 28:e70307. 10.1111/jcmm.70307 39724413 PMC11670868

[B29] Huang Y. L. Zhu Z. Q. (2024). Current status of sevoflurane anesthesia in association with microglia inflammation and neurodegenerative diseases. *iBrain* 10 217–224. 10.1002/ibra.12021 38915946 PMC11193866

[B30] Jangra S. Ashok V. Sethi S. Ram J. (2022). Atomised intranasal dexmedetomidine versus oral melatonin in prevention of emergence delirium in children undergoing ophthalmic surgery with sevoflurane: A randomised double-blind study. *Eur. J. Anaesthesiol.* 39 868–874. 10.1097/EJA.0000000000001727 35875916

[B31] Jia M. Liu W. X. Yang J. J. Xu N. Xie Z. M. Ju L. S. (2016). Role of histone acetylation in long-term neurobehavioral effects of neonatal exposure to sevoflurane in rats. *Neurobiol. Dis.* 91 209–220. 10.1016/j.nbd.2016.03.017 27001149 PMC4860151

[B32] Jia R. Oda S. Yokoi T. (2020). Pharmacological evidence for the involvement of ryanodine receptors in halothane-induced liver injury in mice. *Toxicology* 443:152560. 10.1016/j.tox.2020.152560 32795494

[B33] Jin L. Yu X. Zhou X. Li G. Li W. He Y. (2024). The miR-182-5p/GPX4 pathway contributes to sevoflurane-induced ototoxicity via ferroptosis. *Int. J. Mol. Sci.* 25:6774. 10.3390/ijms25126774 38928480 PMC11204258

[B34] Joe Y. E. Jun J. H. Oh J. E. Lee R. (2024). Damage-associated molecular patterns as a mechanism of sevoflurane-induced neuroinflammation in neonatal rodents. *Korean J. Anesthesiol.* 77 468–479. 10.4097/kja.23796 38556956 PMC11294876

[B35] Joksimovic S. M. Osuru H. P. Oklopcic A. Beenhakker M. P. Jevtovic-Todorovic V. Todorovic S. M. (2018). Histone deacetylase inhibitor entinostat (MS-275) restores anesthesia-induced alteration of inhibitory synaptic transmission in the developing rat hippocampus. *Mol. Neurobiol.* 55 222–228. 10.1007/s12035-017-0735-8 28840475 PMC5808908

[B36] Ju S. Jia M. Sun J. Sun R. Zhang H. Ji H. (2016). Hypermethylation of hippocampal synaptic plasticity-related genes is involved in neonatal sevoflurane exposure-induced cognitive impairments in rats. *Neurotoxicity Res.* 29 243–255. 10.1007/s12640-015-9585-1 26678494

[B37] Kang L. Piao M. Liu N. Gu W. Feng C. (2024). Sevoflurane exposure induces neuronal cell ferroptosis initiated by increase of intracellular hydrogen peroxide in the developing brain via ER stress ATF3 activation. *Mol. Neurobiol.* 61 2313–2335. 10.1007/s12035-023-03695-z 37874483 PMC10972952

[B38] Kong F. L. Wang X. P. Li Y. N. Wang H. X. (2018). The role of exosomes derived from cerebrospinal fluid of spinal cord injury in neuron proliferation in vitro. *Artificial Cells Nanomed. Biotechnol.* 46 200–205. 10.1080/21691401.2017.1304408 28346015

[B39] König M. Lin M. Nelson T. E. Groban L. (2009). Sevoflurane modulation of Ca2+ regulation in skeletal muscle sarcoplasmic reticulum vesicles from young and mature rabbits. *Paediatric Anaesthesia* 19 1166–1174. 10.1111/j.1460-9592.2009.03159.x 19863735

[B40] Kozutsumi Y. Segal M. Normington K. Gething M. J. Sambrook J. (1988). The presence of malfolded proteins in the endoplasmic reticulum signals the induction of glucose-regulated proteins. *Nature* 332 462–464. 10.1038/332462a0 3352747

[B41] Lai R. C. Shan R. Zhou D. Zeng X. Zuo K. Pan D. (2019). Sevoflurane promotes migration, invasion, and colony-forming ability of human glioblastoma cells possibia increasing the expression of cell surface protein 44. *Acta Pharmacol. Sinica* 40 1424–1435. 10.1038/s41401-019-0232-x 30967592 PMC6889382

[B42] Laver D. R. Attia J. Oldmeadow C. Quail A. W. (2017). Cardiac calcium release channel (Ryanodine Receptor 2) regulation by halogenated anesthetics. *Anesthesiology* 126 495–506. 10.1097/ALN.0000000000001519 28079567

[B43] Li P. Liu J. Wang R. Cao F. Li J. Wang H. (2025). Myricetin mitigated sevoflurane-induced cognitive dysfunction in aged mice through inhibiting histone deacetylase 2/nuclear factor erythroid 2-related factor 2/heme oxygenase-1 signaling-mediated ferroptosis and mitochondrial dysfunction. *Mol. Neurobiol.* 62 7776–7791. 10.1007/s12035-025-04703-0 39937417

[B44] Li Q. Q. Yu Q. Liu Z. Y. Zhang Q. Li M. Y. Hu Y. (2025). Sevoflurane anesthesia during late gestation induces cognitive disorder in rat offspring via the TLR4/BDNF/TrkB/CREB pathway. *J. Neuropathol. Exp. Neurol.* 84 244–254. 10.1093/jnen/nlae096 39271176

[B45] Li S. Hou Q. Wang R. Hou Y. Wang Q. Zhang B. (2023). Sevoflurane upregulates neuron death process-related Ddit4 expression by NMDAR in the hippocampus. *Aging* 15 5698–5712. 10.18632/aging.204822 37348034 PMC10333074

[B46] Li Y. Xia H. Chen L. Zhang X. (2017). Sevoflurane induces endoplasmic reticulum stress-mediated apoptosis in mouse hippocampal neuronal HT22 cells via modulating miR-15b-5p/Rab1A signaling pathway. *Int. J. Clin. Exp. Pathol.* 10 8270–8280. 31966678 PMC6965408

[B47] Li Y. Zhang Q. Yu J. Yin C. Zhao J. Wang Q. (2022). Role of BDNF/TrkB signaling pathway in pre-injection of young rat plasma-induced reduction of sevoflurane-caused cognitive dysfunction in aged rats. *Chinese J. Anesthesiol.* 42 546–550.

[B48] Li Z. Wang T. Yu Y. (2022). miR-424 inhibits apoptosis and inflammatory responses induced by sevoflurane through TLR4/MyD88/NF-κB pathway. *BMC Anesthesiol.* 22:52. 10.1186/s12871-022-01590-z 35196982 PMC8864910

[B49] Liang L. Zeng T. Zhao Y. Lu R. Guo B. Xie R. (2021). Melatonin pretreatment alleviates the long-term synaptic toxicity and dysmyelination induced by neonatal Sevoflurane exposure via MT1 receptor-mediated Wnt signaling modulation. *J. Pineal Res.* 71:e12771. 10.1111/jpi.12771 34585785 PMC9285571

[B50] Liu A. Li Y. Tan T. Tian X. (2014). Early exposure to sevoflurane inhibits Ca2+ channels activity in hippocampal ca1 pyramidal neurons of developing rats. *Brain Res.* 1557 1–11. 10.1016/j.brainres.2014.02.008 24518287

[B51] Liu B. Ou G. Chen Y. Zhang J. (2019). Inhibition of protein tyrosine phosphatase 1B protects against sevoflurane-induced neurotoxicity mediated by ER stress in developing brain. *Brain Res. Bull.* 146, 28–39. 10.1016/j.brainresbull.2018.12.007 30553844

[B52] Liu B. Xia J. Chen Y. Zhang J. (2017). Sevoflurane-induced endoplasmic reticulum stress contributes to neuroapoptosis and BACE-1 expression in the developing brain: The role of eIF2a. *Neurotoxicity Res.* 31, 218–229. 10.1007/s12640-016-9671-z 27682474

[B53] Liu D. Jin X. Zhang C. Shang Y. (2018). Sevoflurane relieves hepatic ischemia-reperfusion injury by inhibiting the expression of GRP78. *Biosci. Rep.* 38:BSR20180549. 10.1042/BSR20180549 30217942 PMC6172422

[B54] LiuH.SuB.ZhangZ.JiaS.WangJ.ZhouF. (2025). Neonatal sevoflurane exposures inhibits DHHC5-mediated palmitoylation of TfR1 in oligodendrocytes, leading to hypomyelination and neurological impairments. *J. Adv. Res.* 10.1016/j.jare.2025.02.009 39929269

[B55] Liu T. J. Zhang J. C. Gao X. Z. Tan Z. B. Wang J. J. Zhang P. P. (2018). Effect of sevoflurane on the ATPase activity of hippocampal neurons in a rat model of cerebral ischemia-reperfusion injury via the cAMP-PKA signaling pathway. *Kaohsiung J. Med. Sci.* 34 22–33. 10.1016/j.kjms.2017.09.004 29310813 PMC11915684

[B56] Liu X. Yu J. Tan X. Zhang Q. Niu J. Hou Z. (2024). Necroptosis involved in sevoflurane-induced cognitive dysfunction in aged mice by activating NMDA receptors increasing intracellular calcium. *NeuroToxicology* 100 35–46. 10.1016/j.neuro.2023.12.006 38070654

[B57] Liu Y. Yang H. Tang X. Bai W. Wang G. Tian X. (2016). Repetitive transcranial magnetic stimulation regulates L-type Ca2+ channel activity inhibited by early sevoflurane exposure. *Brain Res.* 1646 207–218. 10.1016/j.brainres.2016.05.045 27256401

[B58] Lv T. Jia F. Wang G. Li S. Wan T. Qiu W. (2024). Sevoflurane causes neurotoxicity and cognitive impairment by regulating hippo signaling pathway-mediated ferroptosis via upregulating PRKCD. *Exp. Neurol.* 377:114804. 10.1016/j.expneurol.2024.114804 38704083

[B59] Lyu N. Li X. (2023). Sevoflurane postconditioning attenuates cerebral ischemia-reperfusion injury by inhibiting SP1/ACSL4-mediated ferroptosis. *Hum. Exp. Toxicol.* 42:9603271231160477. 10.1177/09603271231160477 36842993

[B60] McCann M. E. de Graaff J. C. Dorris L. Disma N. Withington D. Bell G. (2019). Neurodevelopmental outcome at 5 years of age after general anaesthesia or awake-regional anaesthesia in infancy (GAS): An international, multicentre, randomised, controlled equivalence trial. *Lancet* 393 664–677. 10.1016/S0140-6736(18)32485-1 30782342 PMC6500739

[B61] Monk T. G. Weldon B. C. Garvan C. W. Dede D. E. van der Aa M. T. Heilman K. M. (2008). Predictors of cognitive dysfunction after major noncardiac surgery. *Anesthesiology* 108 18–30. 10.1097/01.anes.0000296071.19434.1e 18156878

[B62] Ni H. Chen Y. Xie Y. (2024). Melatonin ameliorates sevoflurane anesthesia-induced deficits in learning and memory of aged mice through Nrf2 signaling related ferroptosis. *Rejuvenation Res.* 27 24–32. 10.1089/rej.2023.0051 38183625

[B63] Ni W. Zou Z. W. Jiang P. Wang S. (2024). Sevoflurane alleviates inflammation, apoptosis and permeability damage of human umbilical vein endothelial cells induced by lipopolysaccharide by inhibiting endoplasmic reticulum stress via upregulating ROR. Prostaglandins other lipid. *Mediat.* 172:106821. 10.1016/j.prostaglandins.2024.106821 38373554

[B64] Niu J. Y. Wu Z. Y. Xue H. Zhang Y. H. Gao Q. S. Li C. (2021). Sevoflurane post-conditioning alleviated hypoxic-ischemic brain injury in neonatal rats by inhibiting endoplasmic reticulum stress-mediated autophagy via IRE1 signalings. *Neurochem. Int.* 150:105198. 10.1016/j.neuint.2021.105198 34601014

[B65] Okuno T. Koutsogiannaki S. Hou L. Bu W. Ohto U. Eckenhoff R. G. (2019). Volatile anesthetics isoflurane and sevoflurane directly target and attenuate toll-like receptor 4 system. *FASEB J.* 33 14528–14541. 10.1096/fj.201901570R 31675483 PMC6894077

[B66] Osman V. Speigel I. Patel K. Hemmings H. C. (2023). Isoflurane alters presynaptic endoplasmic reticulum calcium dynamics in wild-type and malignant hyperthermia-susceptible rodent hippocampal neurons. *eNeuro* 10:ENEURO.114–ENEURO.123. 10.1523/ENEURO.0114-23.2023 37591734 PMC10467020

[B67] Pavlov V. A. Tracey K. J. (2005). The cholinergic anti-inflammatory pathway. *Brain Behav. Immunity* 19 493–499. 10.1016/j.bbi.2005.03.015 15922555

[B68] Pinheiro A. Gomez C. N. Guatimosim R. S. Silva C. Prado J. H. Gomez A. M. (2006). The effect of sevoflurane on intracellular calcium concentration from cholinergic cells. *Brain Res. Bull.* 69 147–152. 10.1016/j.brainresbull.2005.11.016 16533663

[B69] Ren G. Zhou Y. Liang G. Yang B. Yang M. King A. (2017). General anesthetics regulate autophagy via modulating the inositol 1,4,5-trisphosphate receptor: Implications for dual effects of cytoprotection and cytotoxicity. *Sci. Rep.* 7:12378. 10.1038/s41598-017-11607-0 28959036 PMC5620053

[B70] Shan Y. Y. Yang F. Tang Z. Bi C. J. Sun S. W. Zhang Y. F. (2018). Dexmedetomidine ameliorates the neurotoxicity of sevoflurane on the immature brain through the BMP/SMAD signaling pathway. *Front. Neurosci.* 12:964. 10.3389/fnins.2018.00964 30618586 PMC6304752

[B71] Shen F. Y. Lim B.-G. Wen W. Zhang Y. Cao B. Si Y.-G. (2020). Role of T-type calcium channels in generating hyperexcitatory behaviors during emergence from sevoflurane anesthesia in neonatal rats. *Neurosci. Bull.* 36 519–529. 10.1007/s12264-019-00461-x 31953800 PMC7186286

[B72] Shen Q. Jiang Y. Jia X. Zhou X. Zhou Q. (2022). Amelioratory effect of melatonin on cognition dysfunction induced by sevoflurane anesthesia in aged mice. *Iranian J. Pharm. Res.* 21:e133971. 10.5812/ijpr-133971 36896324 PMC9990511

[B73] Shi M. Miao S. Gu T. Wang D. Zhang H. Liu J. (2019). Dexmedetomidine for the prevention of emergence delirium and postoperative behavioral changes in pediatric patients with sevoflurane anesthesia: A double-blind, randomized trial. Drug Design Dev. *Therapy* 13 897–905. 10.2147/DDDT.S196075 30936683 PMC6421876

[B74] Sprung J. Kruthiventi S. C. Warner D. O. Knopman D. S. Petersen R. C. Mielke M. M. (2019). Exposure to surgery under general anaesthesia and brain magnetic resonance imaging changes in older adults. *Br. J. Anaesthesia* 123 808–817. 10.1016/j.bja.2019.08.024 31587833 PMC6883493

[B75] Sun L. S. Li G. Miller T. L. K. Salorio C. Byrne M. W. Bellinger D. C. (2016). Association between a single general anesthesia exposure before age 36 months and neurocognitive outcomes in later childhood. *JAMA* 315 2312–2320. 10.1001/jama.2016.6967 27272582 PMC5316422

[B76] Tang C. Zheng X. Zhong Y. Chen D. Zhu Y. Wang S. (2023). The role of TREM1 in regulating microglial polarization in sevoflurane-induced perioperative neurocognitive disorders. *J. Neuroimmunol.* 379:578070. 10.1016/j.jneuroim.2023.578070 37148600

[B77] Tang W. He D. Liu Y. (2020). Effect of dexmedetomidine in children undergoing general anaesthesia with sevoflurane: A meta-analysis and systematic review. *J. Int. Med. Res.* 48:300060520927530. 10.1177/0300060520927530 32583698 PMC7318832

[B78] Tang X. Li Y. Ao J. Ding L. Liu Y. Yuan Y. (2018). Role of α7nAChR-NMDAR in sevoflurane-induced memory deficits in the developing rat hippocampus. *PLoS One* 13:e0192498. 10.1371/journal.pone.0192498 29401517 PMC5798850

[B79] Tang X. Zhao Y. Zhou Z. Yan J. Zhou B. Chi X. (2020). Resveratrol mitigates sevoflurane-induced neurotoxicity by the SIRT1-dependent regulation of BDNF expression in developing mice. *Oxidative Med. Cell. Longevity* 2020:9018624. 10.1155/2020/9018624 32148659 PMC7049870

[B80] Tian D. Tian M. Ma Z. Zhang L. Cui Y. Li J. (2016). Voluntary exercise rescues sevoflurane-induced memory impairment in aged male mice. *Exp. Brain Res.* 234 3613–3624. 10.1007/s00221-016-4756-8 27540727

[B81] Uryash A. Mijares A. Lopez C. E. Adams J. A. Allen P. D. Lopez J. R. (2024). Post-anesthesia cognitive dysfunction in mice is associated with an age-related increase in neuronal intracellular [Ca2+]-neuroprotective effect of reducing Intracellular [Ca2+]: In vivo and in vitro studies. *Cells* 13:264. 10.3390/cells13030264 38334656 PMC10854970

[B82] Useinovic N. Maksimovic S. Liechty C. Cabrera O. H. Quillinan N. Jevtovic-Todorovic V. (2022). Systemic inflammation exacerbates developmental neurotoxicity induced by sevoflurane in neonatal rats. *Br. J. Anaesthesia* 129 555–566. 10.1016/j.bja.2022.05.008 35701270 PMC10080473

[B83] Wang H. Yu M. Ochani M. Amella C. A. Tanovic M. Susarla S. (2003). Nicotinic acetylcholine receptor Alpha7 subunit is an essential regulator of inflammation. *Nature* 421 384–388. 10.1038/nature01339 12508119

[B84] Wang J. Y. Feng Y. Fu Y. H. Liu G. L. (2018). Effect of sevoflurane anesthesia on brain is mediated by lncRNA HOTAIR. *J. Mol. Neurosci.* 64 346–351. 10.1007/s12031-018-1029-y 29352445

[B85] Wang L. Luo H. Qin G. Cao Y. Gao X. Zhang Z. (2017). The impact of sevoflurane on coupling of the left ventricular-to-systemic vasculature in rats with chronic pulmonary hypertension. *J. Cardiothoracic Vascular Anesthesia* 31 2027–2034. 10.1053/j.jvca.2017.02.049 28533073

[B86] Wang R. Liu P. Li F. Qiao H. (2023). Neuroprotective effect of dexmedetomidine pretreatment on sevoflurane-initiated neurotoxicity Via the miR-204-5p/SOX4 axis. *Protein Peptide Lett.* 30 608–618. 10.2174/0929866530666230530164913 37259215

[B87] Wang T. Weng H. Li Y. (2025). Comparative study of the effects of prenatal sevoflurane exposure at different cortical stages on forebrain development and maturation in offspring. *Front. Neurosci.* 19:1556703. 10.3389/fnins.2025.1556703 40248263 PMC12003305

[B88] Wang T. Wu X. Zhao X. Li J. Yu J. Sheng M. (2024). Sevoflurane Alters serum metabolites in elders and aging mice and increases inflammation in hippocampus. *J. Inflammation Res.* 17 1241–1253. 10.2147/JIR.S448959 38415263 PMC10898602

[B89] Wang Y. Wu D. Li D. Zhou X. Fan D. Pan J. (2023). The role of PERK-eIF2α-ATF4-CHOP pathway in sevoflurane-induced neuroapoptosis and cognitive dysfunction in aged mice. *Cell. Signalling* 110:110841. 10.1016/j.cellsig.2023.110841 37549858

[B90] Wang Z. Zhang J. Tang Q. Tan Y. (2024). Epigenetic mechanism of SETD1B-mediated histone methylation in cognitive impairment induced by sevoflurane anesthesia in neonatal mice. *Neuroscience* 545 1–15. 10.1016/j.neuroscience.2024.02.005 38447691

[B91] Warner D. O. Zaccariello M. J. Katusic S. K. Schroeder D. R. Hanson A. C. Schulte P. J. (2018). Neuropsychological and behavioral outcomes after exposure of young children to procedures requiring general anesthesia: The mayo anesthesia safety in kids (MASK) study. *Anesthesiology* 129 89–105. 10.1097/ALN.0000000000002232 29672337 PMC6008202

[B92] Weiser T. G. Haynes A. Molina G. Lipsitz S. R. Esquivel M. M. Uribe-Leitz T. (2015). Global surgery 2030: Evidence and solutions for achieving health, welfare, and economic development. *Lancet* 386 569–624. 10.1016/j.ijoa.2015.09.006 25924834

[B93] Wu H. Wang S. Dai F. B. Tang C. L. (2025). Research progress in the clinical application of inhaled anesthetic sevoflurane. *Med. Gas Res.* 15 85–92. 10.4103/mgr.MEDGASRES-D-23-00003 39436171 PMC11515067

[B94] Wu J. Yang J. J. Cao Y. Li H. Zhao H. Yang S. (2020). Iron overload contributes to general anaesthesia-induced neurotoxicity and cognitive deficits. *J. Neuroinflammation* 17:110. 10.1186/s12974-020-01777-6 32276637 PMC7149901

[B95] Xiang F. Wang X. Wu Y. Dong N. Sheng Y. (2020). Sevoflurane-induced cognitive decline in aged mice: Involvement of toll-like receptors 4. *Brain Res. Bull.* 165 23–29. 10.1016/j.brainresbull.2020.08.030 32910992

[B96] Xie A. Zhang X. Ju F. Zhou Y. Wu D. Han J. (2024). Sevoflurane impedes neuropathic pain by maintaining endoplasmic reticulum stress and oxidative stress homeostasis through inhibiting the activation of the PLCγ/CaMKII/IP3R signaling pathway. *Aging* 16 11062–11071. 10.18632/aging.206001 38975935 PMC11272110

[B97] Xie L. Fang Q. Wei X. Zhou L. Wang S. (2021). Exogenous insulin-like growth factor 1 attenuates sevoflurane anesthesia-induced cognitive dysfunction in aged rats. *J. Neurophysiol.* 125 2117–2124. 10.1152/jn.00124.2021 33949883

[B98] Xu S. Gao R. Chen L. (2021). Dexmedetomidine regulates sevoflurane-induced neurotoxicity through the miR-330-3p/ULK1 Axis. *J. Biochem. Mol. Toxicol.* 35:e22919. 10.1002/jbt.22919 34590382

[B99] Xu X. Shen X. Wang J. Feng W. Wang M. Miao X. (2021). YAP prevents premature senescence of astrocytes and cognitive decline of Alzheimer’s disease through regulating CDK6 signaling. *Aging Cell* 20:e13465. 10.1111/acel.13465 34415667 PMC8441453

[B100] Xu Y. Wang X. Xu Z. Sun F. Tian Y. (2023). Tbx2 knockdown alleviated sevoflurane-induced cognitive disorder and neuron damages in aged rats via suppressing oxidative stress and ferroptosis. *Toxicol. Sci.* 195 257–269. 10.1093/toxsci/kfad071 37494465

[B101] Xu Y. Zhang N. Chen C. Xu X. Luo A. Yan Y. (2022). Sevoflurane induces ferroptosis of glioma cells through activating the ATF4-CHAC1 pathway. *Front. Oncol.* 12:859621. 10.3389/fonc.2022.859621 35372041 PMC8969566

[B102] Xu Z. Qian B. (2020). Sevoflurane anesthesia-mediated oxidative stress and cognitive impairment in hippocampal neurons of old rats can be ameliorated by expression of brain-derived neurotrophic factor. *Neurosci. Lett.* 721:134785. 10.1016/j.neulet.2020.134785 32027953

[B103] Yang Z. Liu J. Chu H. (2020). Effect of NMDAR-NMNAT1/2 pathway on neuronal cell damage and cognitive impairment of sevoflurane-induced aged rats. *Neurol. Res.* 42 108–117. 10.1080/01616412.2019.1710393 31941414

[B104] Yap J. K. Y. Pickard B. S. Chan E. W. L. Gan S. Y. (2019). The role of neuronal NLRP1 inflammasome in Alzheimer’s disease: Bringing neurons into the neuroinflammation game. *Mol. Neurobiol.* 56 7741–7753. 10.1007/s12035-019-1638-7 31111399

[B105] Yin C. Zhang Q. Zhao J. Li Y. Yu J. Li W. (2022). Necrostatin-1 against sevoflurane-induced cognitive dysfunction involves activation of BDNF/TrkB pathway and inhibition of necroptosis in aged rats. *Neurochem. Res.* 47 1060–1072. 10.1007/s11064-021-03505-9 35040026

[B106] Yin J. Zhao X. Wang L. Xie X. Geng H. Zhan X. (2019). Sevoflurane-induced inflammation development: Involvement of cholinergic anti-inflammatory pathway. *Behav. Pharmacol.* 30 730–737. 10.1097/FBP.0000000000000507 31625977

[B107] You Y. Zhou X. Tang Q. Zhao T. Wang J. Huang H. (2024). Echinatin mitigates sevoflurane-induced neurotoxicity through regulation of ferroptosis and iron homeostasis. *Aging* 16 4670–4683. 10.18632/aging.205622 38446592 PMC10968708

[B108] Zeng F. Zhou M. Li Q. Hu H. Chen C. (2024). Sevoflurane promotes neuronal ferroptosis via upregulation of PLIN4 to modulate the hippo signaling pathway. *NeuroToxicology* 105 1–9. 10.1016/j.neuro.2024.08.001 39182851

[B109] Zeng S. Zhu R. Wang Y. Yang Y. Li N. Fu N. (2022). Role of GABA(A) receptor depolarization-mediated VGCC activation in sevoflurane-induced cognitive impairment in neonatal mice. *Front. Cell. Neurosci.* 16:964227. 10.3389/fncel.2022.964227 36176629 PMC9514857

[B110] Zhang H. Niu Y. Qiu L. Yang J. Sun J. Xia J. (2023). Melatonin-mediated mitophagy protects against long-term impairments after repeated neonatal sevoflurane exposures. *Int. Immunopharmacol.* 125:111210. 10.1016/j.intimp.2023.111210 37976600

[B111] Zhang H. Wu Z. Zhao X. Qiao Y. (2018). Role of dexmedetomidine in reducing the incidence of postoperative cognitive dysfunction caused by sevoflurane inhalation anesthesia in elderly patients with esophageal carcinoma. *J. Cancer Res. Therapeutics* 14 1497–1502. 10.4103/jcrt.JCRT_164_18 30589029

[B112] Zhang P. Chen Y. Zhang S. Chen G. (2022). Mitochondria-related ferroptosis drives cognitive deficits in neonatal mice following sevoflurane administration. *Front. Med.* 9:887062. 10.3389/fmed.2022.887062 35935755 PMC9355652

[B113] Zhang Q. Li Y. Wang X. Yin C. Zhou Q. Guo J. (2022). Sevoflurane exposure causes neuronal apoptosis and cognitive dysfunction by inducing ER stress via activation of the inositol 1,4,5-trisphosphate receptor. *Front. Aging Neurosci.* 14:990679. 10.3389/fnagi.2022.990679 36337694 PMC9631943

[B114] Zhang X. Shen F. Xu D. Zhao X. A. (2016). Lasting effect of postnatal sevoflurane anesthesia on the composition of NMDA receptor subunits in rat prefrontal cortex. *Int. J. Developmental Neurosci.* 54 62–69. 10.1016/j.ijdevneu.2016.01.008 27025552

[B115] Zhang X. Xing Z. Li J. Tang S. Zhang Y. (2021). Gold nanoparticles with dexmedetomidine regulate GSK-3β to reduce neurocognitive effects in anesthetized rats. *J. Nanosci. Nanotechnol.* 21 6205–6211. 10.1166/jnn.2021.18745 34229822

[B116] Zhang Y. Liu X. Xie L. Hong J. Zhuang Q. Ren L. (2024). Overexpression of Nfs1 cysteine desulfurase relieves sevoflurane-induced neurotoxicity and cognitive dysfunction in neonatal mice via suppressing oxidative stress and ferroptosis. *J. Biochem. Mol. Toxicol.* 38:e70051. 10.1002/jbt.70051 39488760

[B117] Zhao D. Zhang M. Yang L. Zeng M. (2022). GPR68 improves nerve damage and myelination in an immature rat model induced by sevoflurane anesthesia by activating cAMP/CREB to mediate BDNF. *ACS Chem. Neurosci.* 13 423–431. 10.1021/acschemneuro.1c00830 35025202

[B118] Zhao J. Ren J. Liu S. Gong Y. Meng P. Tan H. (2021). Repeated exposure to sevoflurane in neonatal rats impairs cognition in adulthood via the PKA-CREB-BDNF signaling pathway. *Exp. Therapeutic Med.* 22:1442. 10.3892/etm.2021.10877 34721684 PMC8549089

[B119] Zhao L. Gong H. Huang H. Tuerhong G. Xia H. (2021). Participation of mind Bomb-2 in sevoflurane anesthesia induces cognitive impairment in aged mice via modulating ferroptosis. *ACS Chem. Neurosci.* 12 2399–2408. 10.1021/acschemneuro.1c00131 34121396

[B120] Zhao Z. Ma L. Li Y. Zhang Q. Wang Y. Tai Y. (2021). MiR-124 protects against cognitive dysfunction induced by sevoflurane anesthesia in vivo and in vitro through targeting calpain small subunit 1 via NF-κB signaling pathway. *Adv. Clin. Exp. Med.* 30 701–709. 10.17219/acem/134740 34118141

[B121] Zhu G. Tao L. Wang R. Xue Y. Wang X. Yang S. (2017). Endoplasmic reticulum stress mediates distinct impacts of sevoflurane on different subfields of immature hippocampus. *J. Neurochem.* 142 272–285. 10.1111/jnc.14057 28444766

[B122] Zuo C. Ma J. Pan Y. Zheng D. Chen C. Ruan N. (2022). Isoflurane and sevoflurane induce cognitive impairment in neonatal rats by inhibiting neural stem cell development through microglial activation, neuroinflammation, and suppression of VEGFR2 signaling pathway. *Neurotoxicity Res.* 40 775–790. 10.1007/s12640-022-00511-9 35471722 PMC9098611

